# Crystalline silica-induced recruitment and immuno-imbalance of CD4^+^ tissue resident memory T cells promote silicosis progression

**DOI:** 10.1038/s42003-024-06662-z

**Published:** 2024-08-09

**Authors:** Yichuan You, Xiulin Wu, Haoyang Yuan, Yangyang He, Yinghui Chen, Sisi Wang, Hui Min, Jie Chen, Chao Li

**Affiliations:** 1 Key Laboratory of Environmental Stress and Chronic Disease Control & Prevention (China Medical University), Ministry of Education, No. 77 Puhe Road, Shenyang North New Area, Shenyang, 110122 Liaoning PR China; 2 Department of Occupational and Environmental Health, School of Public Health, China Medical University, No. 77 Puhe Road, Shenyang North New Area, Shenyang, 110122 Liaoning PR China; 3 Department of Immunology, College of Basic Medical Sciences, China Medical University, No. 77 Puhe Road, Shenyang North New Area, Shenyang, 110122 Liaoning PR China

**Keywords:** Chronic inflammation, Inflammatory diseases, CD4-positive T cells

## Abstract

Occupational crystalline silica (CS) particle exposure leads to silicosis. The burden of CS-associated disease remains high, and treatment options are limited due to vague mechanisms. Here we show that pulmonary CD4^+^ tissue-resident memory T cells (T_RM_) accumulate in response to CS particles, mediating the pathogenesis of silicosis. The T_RM_ cells are derived from peripheral lymphocyte recruitment and in situ expansion. Specifically, CD69^+^CD103^+^ T_RM_-Tregs depend more on circulating T cell replenishment. CD69 and CD103 can divide the T_RM_ cells into functionally distinct subsets, mirroring the immuno-balance within CD4^+^ T_RM_ cells. However, targeting CD103^+^ T_RM_-Tregs do not mitigate disease phenotype since the T_RM_ subsets exert immunosuppressive but not pro-fibrotic roles. After identifying pathogenic CD69^+^CD103^-^ subsets, we highlight IL-7 for their maintenance and function, that present a promising avenue for mitigating silicosis. Together, our findings highlight the distinct role of CD4^+^ T_RM_ cells in mediating CS-induced fibrosis and provide potential therapeutic strategies.

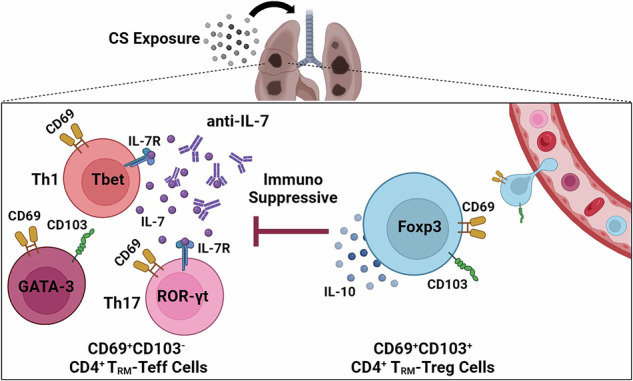

## Abstract (web summary)

Crystalline silica exposure led to CD4^+^ tissue-resident memory T-cell accumulation. The imbalance of pulmonary T_RM_-Teffs and T_RM_-Tregs promoted silicosis progression. Neutralizing IL-7 alleviated silicosis by disrupting T_RM_-Teff maintenance.

## Introduction

Crystalline silica (CS) is a typical inorganic particle in natural and industrial settings. Exposure to respirable CS leads to pneumoconiosis, characterized by chronic inflammation and progressive pulmonary fibrosis^1,2^. Though redoubled efforts were made to minimize CS exposure, stubbornly high morbidity and mortality of silicosis emphasized the hazardous burden of CS-related diseases^3,4^. The new emerging industries like sandblasting denim jeans and manufacturing of artificial stone benchtops reignited the emergence of silicosis around the world^5^. While silicosis is a preventable disease, unfortunately, patients continue to suffer from this progressive disease^2^. Interventions against silicosis progression are in high demand.

The inhaled CS particles deposited in the lung interstitium trigger inflammatory cascades involving innate and adaptive immune responses^6^. Although some early events, such as macrophages engulfing CS particles, in silicosis are clear, the following steps leading to fibrosis are less well-understood^7–9^. Different from the simple exposure-response relationship, adaptive immune response characterized as disorders of T lymphocytes orchestrate chronic inflammation and fibrogenesis^10^. CD4^+^ helper T (Th) cells have been identified as vital players in fibrotic disorders, including silicosis^11^. The CD4^+^ Th cells can be divided into pathogenic effector T cells (including Th1, Th2, and Th17 cells) and immunosuppressive regulatory T cells (Tregs), whose fate was governed by transcriptional factors T-bet, GATA-3, ROR-γt, and FOXP3, respectively^12^. Notably, T cell-mediated adaptive immune response is characterized as immunological memory. Memory T cells (T_M_) expressed memory-T-cell-associated molecule, CD44, and can be divided into several subsets^13^. Central memory T cells (T_CM_) patrol the blood and secondary lymphoid organs, while effector memory T cells (T_EM_) express homing molecules, allowing them access to peripheral tissues^14,15^. The T_CM_ and T_EM_ cells are abundant in circulation, while tissue-resident memory T cells (T_RM_) preferentially localize in barrier tissues such as the lung^16,17^. T_RM_ cells rapidly respond to the invading pathogen within peripheral tissues, providing first-time and robust protection, while in some chronic inflammatory diseases, T_RM_ cells exert pathogenic roles^18^. Th cell’s function in silicosis was explored^19^. However, these researches were largely based on the evidence of peripheral T cells. The knowledge about the contribution of lung resident T_RM_ cells to silicosis is limited.

T cells migrate to the damaged tissue, mature, and maintain in non-lymphoid tissues, exerting enhanced effector functions compared to their lymphoid tissue counterparts. They rapidly respond to the invading pathogen within peripheral tissues, providing first-time and robust protections upon cognate antigen stimulation^20^. Though T_RM_ cells have primarily been described as their protective functions, particularly in the context of pathogen infections, T_RM_ cells, especially CD4^+^ T_RM_ cells, have also been reported to be pathogenic in chronic inflammatory settings^16,21^.

In the pathogenesis of silicosis, the inhaled CS particles can be engulfed by macrophages but cannot be cleared, resulting in the re-release of CS particles, which leads to repeatedly, locally re-initiated chronic inflammation. Under such circumstances, inflammatory cytokines (like IL-17A, IFN-γ, and TNF-α) and profibrotic cytokines (like TGF-β and PDGFα) could promote fibroblast activation, shown by increased fibrosis-related protein, collagen I and fibronectin^22^. Additionally, our previous study demonstrated that cytokine-producing CD4^+^ T cells in the silicotic lung manifested T_M_ cell characterization^23^. Given the situation of repeated CS stimulation and the immunologic memory function of T_RM_ cells, we hypothesized that CD4^+^ T_RM_ cells are involved in the pathogenesis of silicosis.

To this end, we utilized a murine model of silicosis delineating the effects of CS particles on CD4^+^ T_RM_ cells and further explored its pathogenic role in silicosis. Our results demonstrated CS particles induced substantial accumulations of pulmonary CD4^+^ T_RM_ cells. We further proved the source and function of CD4^+^ T_RM_ cells in the pathogenesis of silicosis. The CD69^+^CD103^–^ CD4^+^ T_RM_ subset manifested robust pro-inflammatory responses, whereas the CD69^+^CD103^+^ CD4^+^ T_RM_ subset was immuno-suppressive. Significantly, targeting the maintenance and function of pathogenic lung CD4^+^ T_RM_ cells exerted protective effects against silicosis.

## Results

### CS particles stimulated CD4^+^ T_RM_ cell emergence and expansion along with silicosis progression

**Fig. 1:**
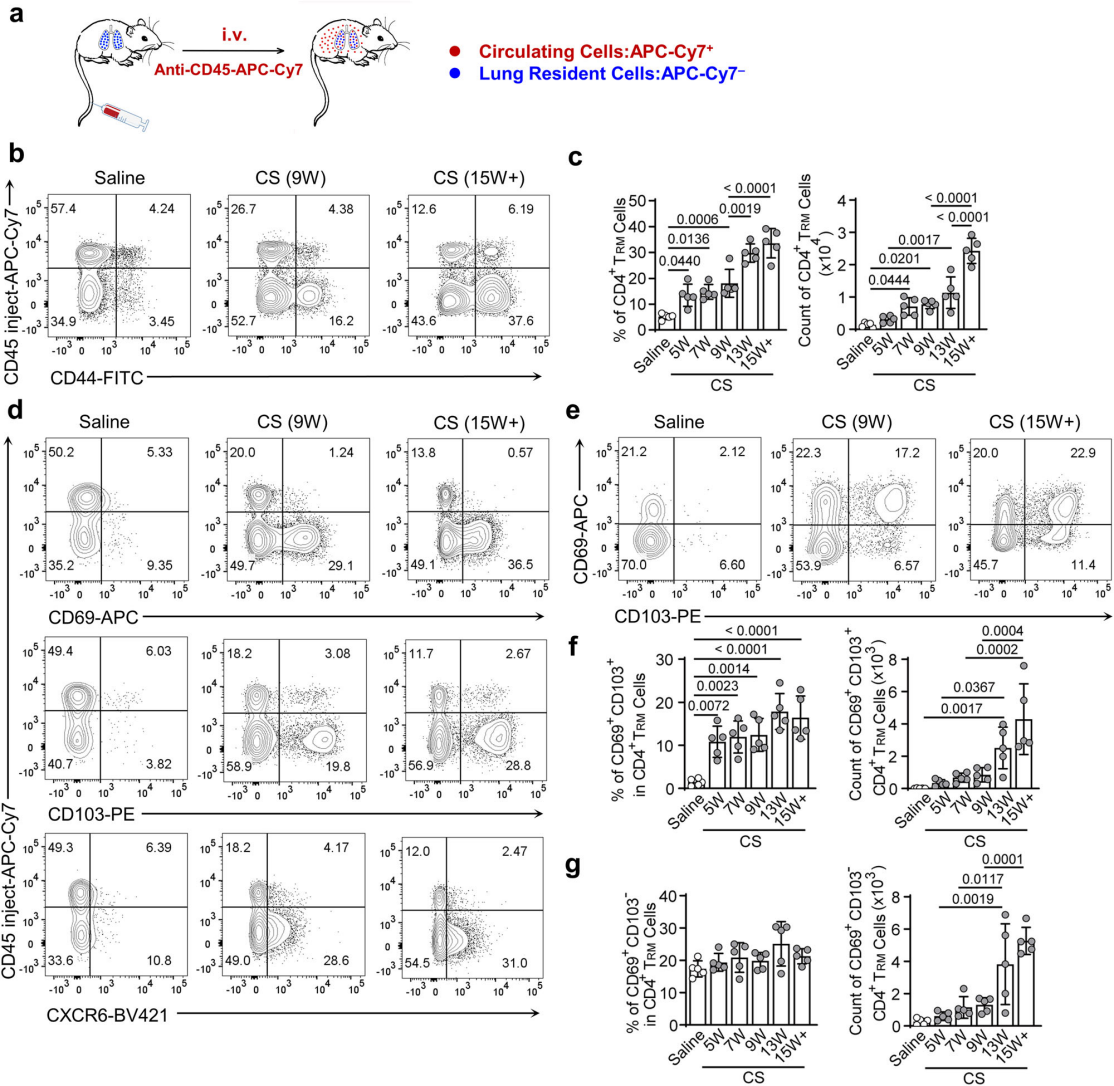
CS particles stimulated CD4^+^ T_RM_ cell emergence and expansion along with silicosis progression. **a** Schematic showed CD45-APC-Cy7 antibody intravenous (i.v.) injection to distinguish tissue-resident or circulating leukocytes. Circulating leukocytes were labeled. **b** Flow cytometry (FC) analysis of pulmonary CD4^+^ T_RM_ cells (CD45_inject_^–^ CD44^+^) and T_EM_ (CD45_inject_^+^ CD44^+^) cells. **c** Percentages and counts of the CD4^+^ T_RM_ cells were compared at the indicated time points. W week. **d** Representative FC analysis of CD4^+^ T_RM_ cells for CD69, CD103, and CXCR6 expression, respectively. **e** FC analysis of CD4^+^ T_RM_ cells for CD69 and CD103 expression. **f**, **g** The graph showed percentages and counts of CD69^+^ CD103^+^ (**f**) and CD69^+^ CD103^–^ subsets (**g**) in CD4^+^ T_RM_ cells of saline or CS-treated mice at the specified time points. The bar graphs are the combined results of at least three independent experiments. Individual mice are plotted on the graphs, *n* = 5 biologically independent animals. Values are reported as the mean ± SD. *P* value was determined by one-way ANOVA followed by Tukey’s test.

First, we utilized the in vivo labeling method distinguishing tissue-resident cells that are commonly used in multi-vascular tissues (Fig. 1a)^23^. We observed a remarkable appearance of CD4^+^ T_RM_ cells (CD44^+^CD45 i.v.^–^) in the silicotic lung (Fig. 1b), whereas few CD4^+^ T_RM_ cells were found in saline-treated mice. Unlike the circulating CD4^+^ T_EM_ cells (CD44^+^CD45 i.v.^+^), CD4^+^ T_RM_ cells surged continuously with the progression of silicosis (Fig. 1c and Fig. S1b), suggesting a link between the emergence of CD4^+^ T_RM_ cells and silicosis progression. Furthermore, compared with saline-treated mice pulmonary CD4^+^ T_RM_ cells, the elevated expressions of cell retention markers, CD69, CD103, and CXCR6 further confirmed silicotic CD4^+^ T_RM_ cells possessed lung retention ability (Fig. 1d). Furthermore, the CD69^+^CD103^–^ subset was observed in both saline- or CS-treated lung, whereas the CD69^+^CD103^+^ subset was distinct in silicotic lung (Fig. 1e). With the expansion of CD4^+^ T_RM_ cells, there was an increasing number of CD69^+^CD103^+^ and CD69^+^CD103^–^ subsets (Fig. 1f, g), implying their pathogenic roles in silicosis. Comparatively, the phenotype of circulating CD4^+^ T_EM_ cells was analogic in saline and CS-treated mice, which differed from T_RM_ cells (Fig. S1c). Notably, CD69 and CD103 expressions on splenic CD4^+^ T_EM_ cells were not affected by CS injury in the lung (Fig. S1d), indicating CS led to a tissue-local response. Collectively, these data demonstrated CS stimulated the emergence and expansion of pulmonary CD4^+^ T_RM_ cells that were tightly related to silicosis progression.

### Pulmonary CD4^+^ T_RM_ cells mediated severe lung inflammatory response to CS particles, promoting the pathogenesis of silicosis

**Fig. 2:**
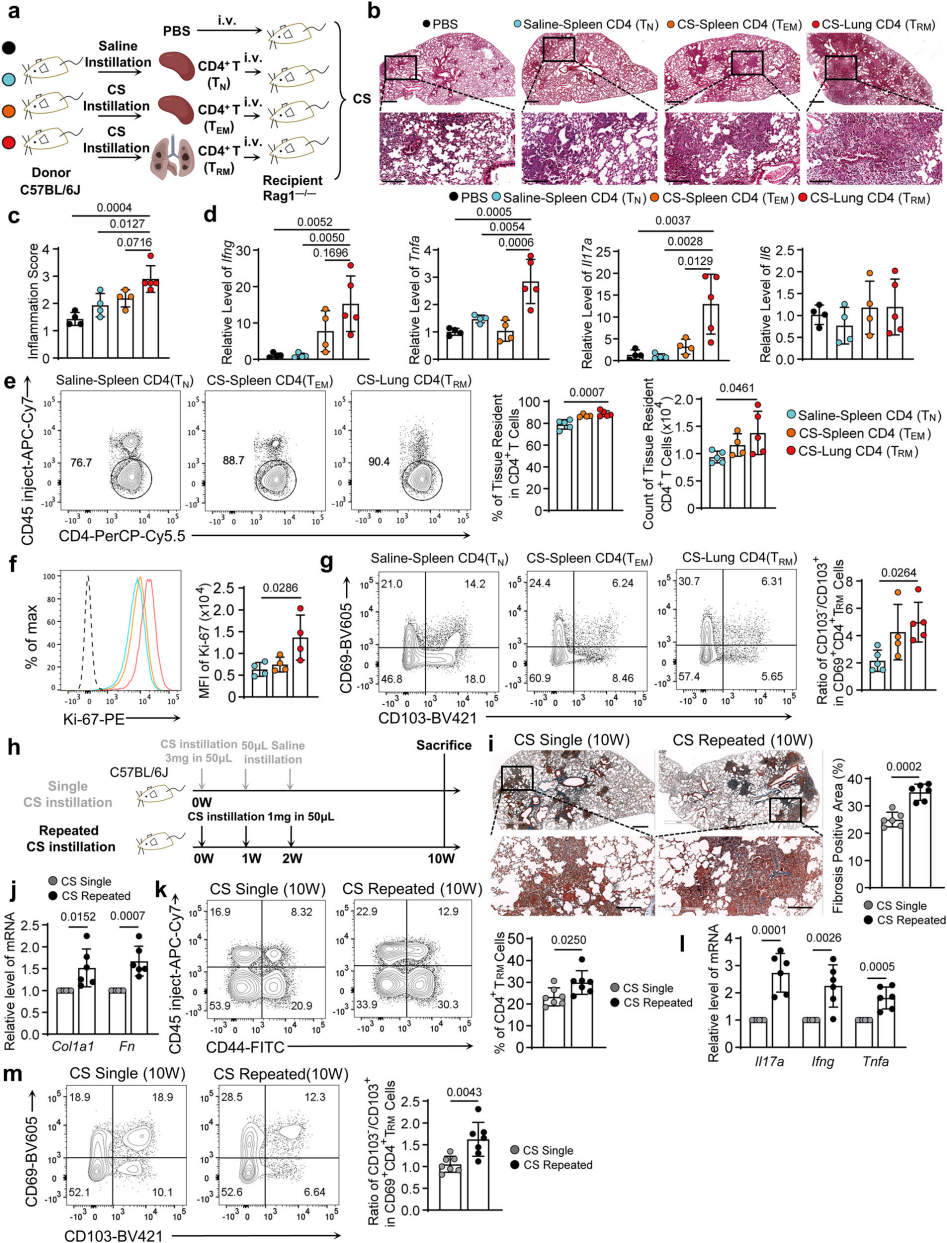
Pulmonary CD4^+^ T_RM_ cells mediated severe lung inflammatory response to CS particles. **a** The reconstitution sketch of *Rag1*^–/–^ mice with different cell origins. The MACS-purified CD4^+^ T cells and PBS were i.v. transferred into *Rag1*^–/–^ mice before CS instillation. The recipient mice were analyzed one week after the CS instillation. **b** H&E staining to the lung sections of distinct reconstituted *Rag1*^–/–^ mice. Scale Bar = 500 μm (upper) and 200 μm (lower). **c** The inflammation scores were assessed in the lung sections. **d** Relative RNA levels of *Ifng*, *Tnfa*, *Il17a*, and *Il6* in each group. **e** FC analysis of pulmonary resident CD4^+^ T cells in transferred *Rag1*^–/–^ mice. **f** Flow histogram indicated Ki-67 intensity in lung-resident CD4^+^ T cells. **g** FC analysis compared the proportions of CD103^–^ to CD103^+^ in the CD69^+^ CD4^+^ T_RM_ cells. **h** The scheme indicated the time points of CS instillation: Single CS instillation was treated at 0 W (3 mg in 50 μL), while 50 μL sterile saline per week was instilled at 1 and 2 weeks. Repeated CS instillation was treated at 0, 1, and 2 W (1 mg in 50 μL per week), respectively. Both groups were sacrificed 10 W after the first CS treatment, with an equal amount of CS particles in total. **i** Masson’s trichrome staining to the lung sections of different groups. Scale bar = 500 and 200 μm. The bar graph showed the fibrosis-positive area. **j** Relative mRNA levels of *Col1a1* and *Fn* in each group. **k** FC analysis of pulmonary CD4^+^ T_RM_ cells in single or repeated CS-treated mice. **l** Relative mRNA levels of *Il17a*, *Ifng*, and *Tnfa* in each group. **m** FC analysis showed the ratios of CD103^–^ to CD103^+^ subsets in CD69^+^ CD4^+^ T_RM_ cells. For panels **a**–**g**, *n* = 4–5 biologically independent animals, *n* number also indicated independent experimental replicates. Littermate recipient *Rag1*^–/–^ mice and donor C57BL/6J mice were used in these experiments. Values are reported as mean ± SD. *P* value was determined by one-way ANOVA followed by Tukey’s test. For panels, **h**–**m**, *n* = 6–7 biologically independent animals, the bar graphs are the combined results of at least three independent experiments. Individual mice are plotted on the graphs. Values are reported as mean ± SD. *P* value was determined by unpaired two-tailed Student’s *t*-test.

Given the immunologic memory role of T_RM_ cells, we next sought to explore the response of CD4^+^ T_RM_ cells to CS particles by T-cell transfer studies. CD4^+^ T cells were sorted from the 8 weeks post-CS-treated lung containing certain T_RM_ cells (T_RM_), the spleen under CS treatment only involving circulating T_EM_ cells (T_EM_), or the spleen of saline-treated mice, including naive T cells (T_N_) to CS particles, respectively. The sorted cells were adoptively transferred into *Rag1*^–/–^ mice that lack T cells, and then all *Rag1*^–/–^ mice were treated with CS (Fig. 2a). H&E staining revealed that CS-induced the severest inflammatory cell recruitment and infiltration in the mice lung transferred with T_RM_ cells. In contrast, the mice who transferred T_N_ or T_EM_ cells exerted relatively mild responses (Fig. 2b, c). These phenotypes were further confirmed by the transcripts of cytokines associated with adaptive immunity, including *Ifng*, *Tnfa*, and *Il17a*, but not *Il6* (Fig. 2d). We further adopted flow analysis to the lungs. Significantly, more pulmonary resident T cells were observed in the mice transferred with CD4^+^ T_RM_ than those of naive counterparts (Fig. 2e), highlighting a rapid reaction and expansion of the CD4^+^ T_RM_ cells to CS particles. In supporting the notion, a higher Ki67, cell proliferating marker, was observed in the cells from the mice transferred with CD4^+^ T_RM_ (Fig. 2f). While lung resident T cells in the mice transferred with CD4^+^ T_EM_ cells resembled those transferred with certain CD4^+^ T_RM_ cells (Fig. 2e), implying the ability of T_EM_ cells converting into T_RM_ cells. Additionally, we discovered the ratio of CD103^–^ to CD103^+^ in CD69^+^ T_RM_ was affected by the distinct cellular sources, while more CD69^+^CD103^–^ subsets resided in the CD4^+^ T_RM_ transferred mice (Fig. 2g). These results implied the CD69^+^CD103^–^ T_RM_ cells mediated pro-inflammatory effects to the invaded CS particles.

Considering persistent particle exposure in the working environments^24,25^, we adopted a repeated CS exposure model (Fig. 2h), exploring the role of CD4^+^ T_RM_ cells in mediating silicosis. Though the mice were exposed to the same amount of CS particles in total, repeated exposure resulted in severe pulmonary fibrosis, which was demonstrated by Masson’s trichrome staining and elevated *Col1a1* and *Fn* transcripts (Fig. 2i, j). We further did flow analysis to the CS-exposed lung, demonstrating that the repeated CS exposure surged more CD4^+^ T_RM_ cells in the lung than the one-time challenge (Fig. 2k). The transcripts of proinflammatory cytokines *Il17a*, *Ifng*, and *Tnfa* were also higher in the CS-repeated exposed lung (Fig. 2l). Precisely, we dissected that there were more CD69^+^CD103^–^ subsets in the CD4^+^ T_RM_ cells (Fig. 2m), which implied repeated CS stimulated specific T_RM_ subsets expansion. In synthesis, these results demonstrated that CD4^+^ T_RM_ cells exerted immuno-memory to the CS particles mediated the pathogenesis of silicosis, and the CD69^+^CD103^–^ T_RM_ subsets possessed robust pathogenic capacity to silicosis.

### CS-induced T_RM_ cells derived from circulating T cell recruitment and proliferation in situ

**Fig. 3:**
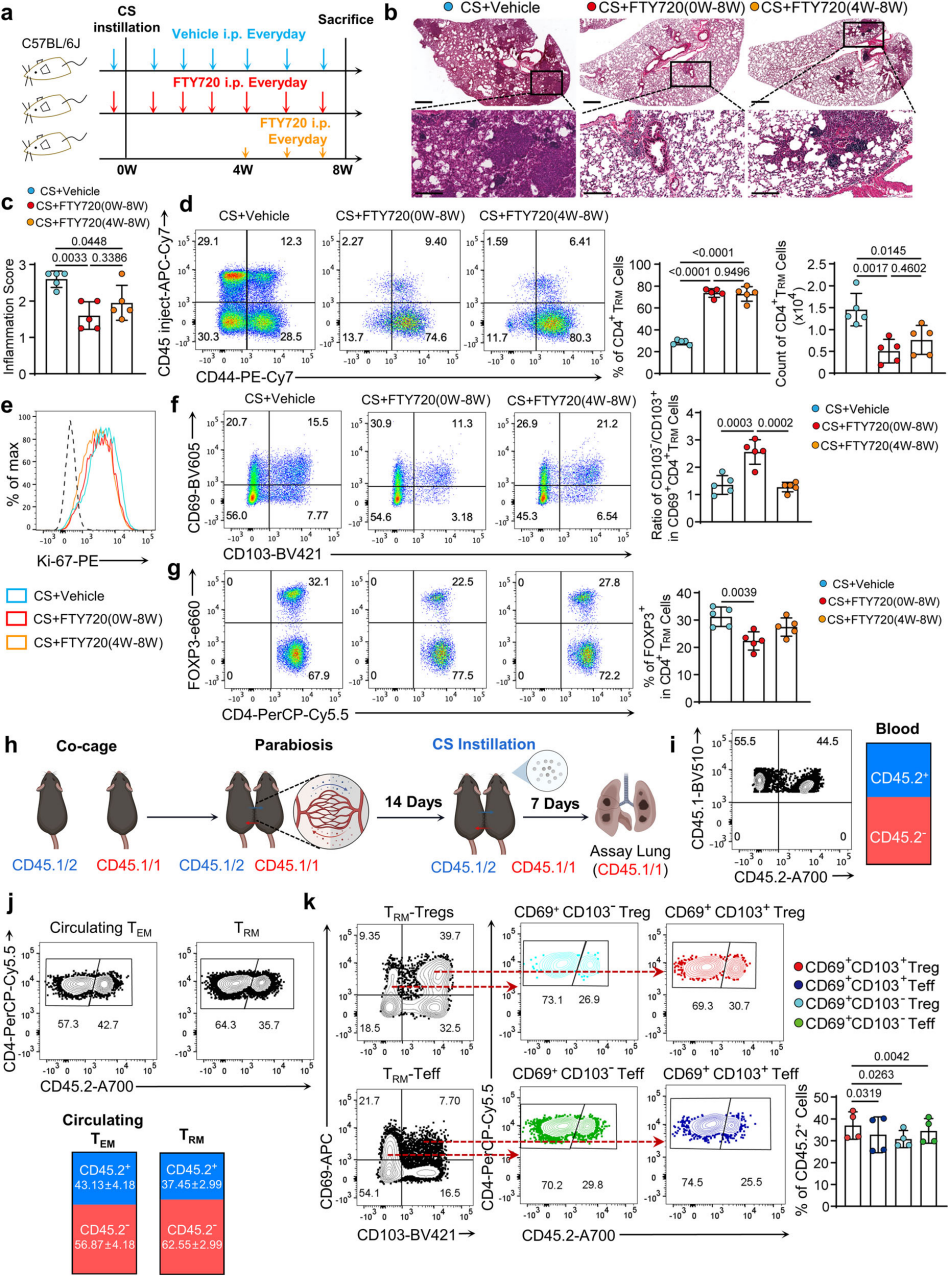
CS-induced T_RM_ cells derived from circulating lymphocyte recruitment and in situ proliferation. **a** The scheme indicated the time points of FTY720 treatment (20 μg in vehicle per time). FTY720 treatment from 4 weeks, in which circumstance, lymphocytes could be recruited into the lung at the inflammatory stage but blocked at the fibrogenesis stage. **b** H&E staining to the lung section of different treated mice. Scale Bar = 500 μm (upper) and 200 μm (lower). **c** The inflammation scores were assessed in the lung sections. **d** FC analysis of CD4^+^ T_RM_ cells in the lungs of distinct FTY720-challenge. The bar graph shows percentages and counts of CD4^+^ T_RM_ cells. **e** Flow histogram indicated Ki-67 expression in CD4^+^ T_RM_ cells. **f** FC analysis of CD4^+^ T_RM_ cells for CD69 and CD103 expressions. The bar graph displayed the ratios of CD103^–^ to CD103^+^ in the CD69^+^ T_RM_ cells. **g** FC analysis of Tregs (FOXP3^+^) in the CD4^+^ T_RM_ cells. The bar graph shows the percentages of T_RM_-Tregs. **h** Schematic overview of parabiosis experiment. CD45.1/1 and CD45.1/2 mice were approximated with sutures. With 14 days’ recovery, CD45.1/1 mice were treated by CS particle instillation and analyzed 7 days later. **i** Typical FC plot of circulating blood leukocytes of the parabiont. **j** Flow plot analysis of the CD4^+^ T_RM_ cells and circulating T_EM_ cells of the CS-treated conjoined mice. CD45.2^+^ T_RM_ cells indicated the recruited cells from circulating. **k** Typical flow plots showed CD69 and CD103 expressing patterns on T_RM_-Teff cells or T_RM_-Tregs. Flow plot analysis of CD45.2^+^ cells in CD69^+^CD103^+^ Tregs, CD69^+^CD103^+^ Teffs, CD69^+^CD103^-^ Tregs, and CD69^+^CD103^-^ Teffs in CD4^+^ T_RM_ cells. For panels **a**–**g**, *n* = 5 biologically independent animals, *n* number also indicated independent experimental replicates. Individual mice are plotted on the graphs. Values are reported as the mean ± SD. *P* value was determined using one-way ANOVA followed by Tukey’s test. For panels **j** and **k**, *n* = 4 biologically independent animals, *n* number also indicated independent experimental replicates. CD45.1/1 and CD45.1/2 mice approximated with sutures were used in these experiments. Values are reported as the mean ± SD. *P* value was determined by paired two-tailed Student’s *t*-test.

We next sought to study the origin of CD4^+^ T_RM_ cells in silicotic lungs. By applying FTY720 treatment in C57BL/6J mice, we blocked leukocyte egress from the peripheral lymphoid tissue, minimizing the recruitment of circulating leukocytes^26^. Particularly, the treatments at different time points within silicosis progression were employed to elucidate the origin and maintenance of CD4^+^ T_RM_ cells (Fig. 3a). H&E staining demonstrated that FTY720 treatment exerted protective effects on silicosis. However, though half-time intervention (4–8 weeks) reduced inflammatory cell recruitment, we could not get an equal attenuated phenotype compared to full-time blockage (0–8 weeks) (Fig. 3b, c), suggesting that peripheral circulating cells would turn into T_RM_ cells. Full-time and half-time FTY720 treatment resulted in a significant reduction of circulating CD4^+^ T cells shown by in vivo labeling. On the contrary, the existence of T_RM_ cells implied that CS-induced pulmonary T_RM_ cells actively expanded in situ (Fig. 3d). Specifically, full-time and half-time FTY720 treatment resulted in an equal vanish of CD4^+^ T_RM_ cells in number, indicating that the recruitment after 4 weeks post-CS-treatment was essential for CD4^+^ T_RM_ cells. Accordingly, high levels of Ki-67 were observed in the T_RM_ cells but not impaired by FTY720 treatments (Fig. 3e). Notably, we noticed affected ratios of CD103^–^ to CD103^+^ in CD69^+^ T_RM_ by the FTY720 intervention that full-time FTY720 treatment resulted in a high portion of CD69^+^CD103^–^ T_RM_ subsets (Fig. 3f) that analog to the phenotypes of previous transfer experiments. We next explored the proportion of Tregs in the T_RM_ cells and got a lower ratio in the full-time FTY720-treated mice lung (Fig. 3g), reminding us that T_RM_-Tregs were more dependent on the replenishing of peripheral circulating cells.

We further performed a parabiosis surgery to illuminate the source of the T_RM_ cells in the CS-injured lung^26,27^. To do this, naive CD45.1/2 mice were cojoined with naive congenic mice (CD45.1/1) (Fig. 3h). The blood circulation between parabionts was established 14 days after the surgery, indicated by an equal portion of CD45.1 and CD45.2 lymphocytes in the blood (Fig. 3i), after which the CD45.1/1 congenic mice were exposed to CS particles. 7 days later, we checked the component of circulating T_EM_ cells and the pulmonary T_RM_ in the CS-exposed mice. We found the composition of CD45.2^+^ lymphocytes in circulating T_EM_ cells was equal to those in the blood (Fig. 3j). Notably, we found that there were CD45.2^+^ cells emergence in the CS-treated CD45.1/1 mice pulmonary T_RM_ cells (Fig. 3j), suggesting circulating T cells contributed to T_RM_ cells. Moreover, we found both the T_RM_-Teff cells and T_RM_-Tregs were dependent on circulating T cells, manifested by a portion of CD45.2^+^ T cells in the congenic mice (Fig. 3k). We also observed that T_RM_-Tregs expressed higher CD103 than the T_RM_-Teff cells (Fig. 3k), which reminded us that the adhesive molecule CD103 was related to distinguish different CD4^+^ T_RM_ subsets. However, when we scrutinized T_RM_-Tregs, we found the highest portion of CD45.2^+^ cells in the CD69^+^CD103^+^ T_RM_-Tregs, proving that CD103^+^ T_RM_-Tregs are dependent more on circulating cell replenishment (Fig. 3k). Collectively, these data demonstrated that CS-induced pulmonary CD4^+^ T_RM_ cells came in two ways: recruited from circulation and proliferating in situ. The T_RM_-Tregs were dependent more on replenishment from circulating cells.

### Differential CD69 and CD103 expressing patterns defined silicotic CD4^+^ T_RM_ cells into relatively functional distinct lineages

**Fig. 4:**
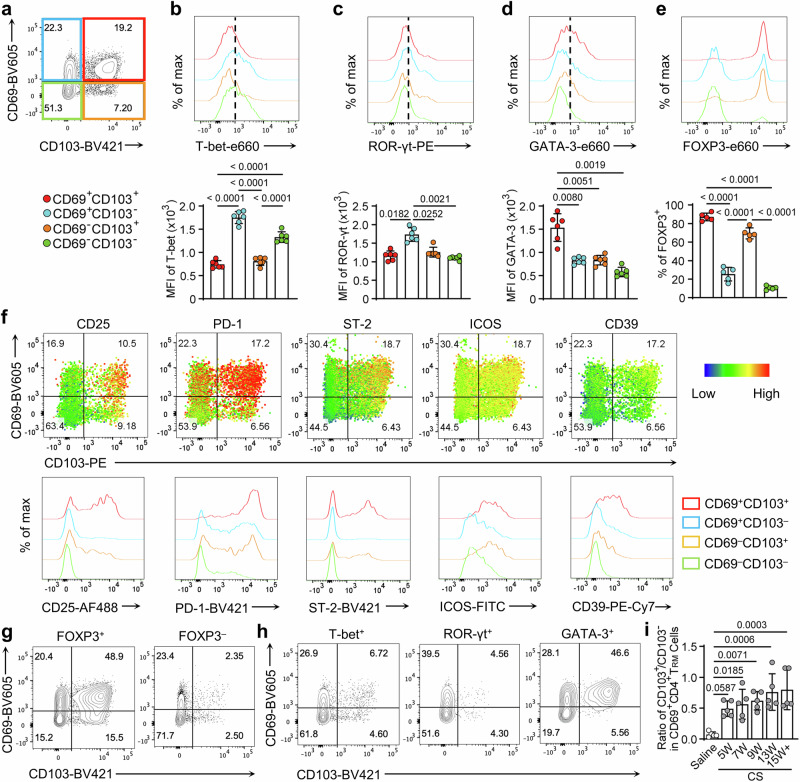
Differential CD69 and CD103 expressions defined silicotic CD4^+^ T_RM_ cells into effector or regulatory T_RM_ cells. **a** Flow plot indicated CD4^+^ T_RM_ cells in fibrotic lungs were divided into four subsets by CD69 and CD103 expressions. CD69^+^CD103^+^ (Red); CD69^+^CD103^–^ (Blue); CD69^–^CD103^+^ (Orange); CD69^–^CD103^–^ (Green). **b**–**d** Flow histogram displayed transcriptional factor T-bet (**b**), ROR-γt (**c**), and GATA-3 (**d**) expressions among different subsets. Bar graph below shows the MFI among the indicated CD4^+^ T_RM_ cell subsets. **e** Flow histogram compared ratios of FOXP3^+^ among different subpopulations. Bar graph below reveals the percentages of FOXP3^+^ among the indicated subsets. **f** FC heatmaps show the expression intensity of each marker. Representative flow histogram compared the intensity of CD25, PD-1, ST2, ICOS, and CD39 among four subsets in CD4^+^ T_RM_ cells. **g** Typical flow plots showed CD69 and CD103 expressing patterns on Tregs (FOXP3^+^) or Teff cells (FOXP3^–^) in CD4^+^ T_RM_ cells. **h** Typical flow plots demonstrated CD69 and CD103 expressing patterns on T-bet^+^, ROR-γt^+^, or GATA-3^+^ subsets in CD4^+^ T_RM_ cells. **i** The bar graph showed the ratios of CD103^+^ against CD103^–^ subsets in CD69^+^ CD4^+^ T_RM_ cells. Bar graphs are the combined results of at least three independent experiments. Individual mice are plotted on the graphs, *n* = 5–6 biologically independent animals. Values are reported as the mean ± SD. *P* value is determined by one-way ANOVA followed by Tukey’s test.

Previous results suggested that adhesion molecule expressions may be related to the constitution of T_RM_ subsets. Next, we aimed to scrutinize the phenotype of CD4^+^ T_RM_ cells in silicotic lungs. The CD4^+^ T_RM_ cells were divided into 4 subsets on differential CD69 and CD103 expressions^28^ (Fig. 4a). Interestingly, the CD69^+^CD103^–^ and CD69^–^CD103^–^ subpopulations expressed higher T-bet, indicating pro-inflammatory Th1 cells were enriched (Fig. 4b). The comparison of ROR-γt manifested that the CD69^+^CD103^–^ subpopulation expressed the highest level (Fig. 4c). The CD69^+^CD103^+^ subset exerted the highest GATA-3 expression (Fig. 4d). A higher portion of FOXP3^+^ was observed in CD69^+^CD103^+^ and CD69^–^CD103^+^ subsets (Fig. 4e). Cell retention markers could depict T_RM_ into relatively distinct subsets. Tregs were enriched in CD103^+^ subsets. In line with this, Tregs’ functional markers, CD25 (IL-2Rα), PD-1, ST2 (IL-33R), ICOS (CD278), and CD39 were highly expressed within the CD103^+^CD69^+^ subset^28^ (Fig. 4f).

To corroborate the findings, we further divided the CD4^+^ T_RM_ cells into Tregs and Teff cells, then checked their CD103 and CD69 expression patterns. Expectedly, T_RM_-Tregs (FOXP3^+^) expressed high CD103, while the T_RM_-Teff cells (FOXP3^–^) expressed few CD103 (Fig. 4g), suggesting CD103 is a good indicator of T_RM_-Tregs. Furthermore, we discovered the Th1-type T_RM_ (T-bet^+^) and Th17-type T_RM_ (ROR-γt^+^) expressed less CD103, whereas Th2-type T_RM_ (GATA-3^+^) expressed relatively high CD103 (Fig. 4h), implying the emergence of GATA-3^+^ Tregs^29^. Remarkably, the ratio of CD103^+^ to CD103^–^ in CD69^+^CD4^+^ T_RM_ cells was lifted with silicosis progression, emphasizing the immune imbalance within T_RM_ subsets would be related to fibrogenesis (Fig. 4i). In synopsis, we proved the cell retention markers CD103 and CD69 can define CD4^+^ T_RM_ cells into functional distinct lineages, which may provide potential therapeutic targets for alleviating silicosis.

### Targeting CD103^+^ T_RM_-Tregs could not mitigate CS-induced pulmonary fibrosis

**Fig. 5:**
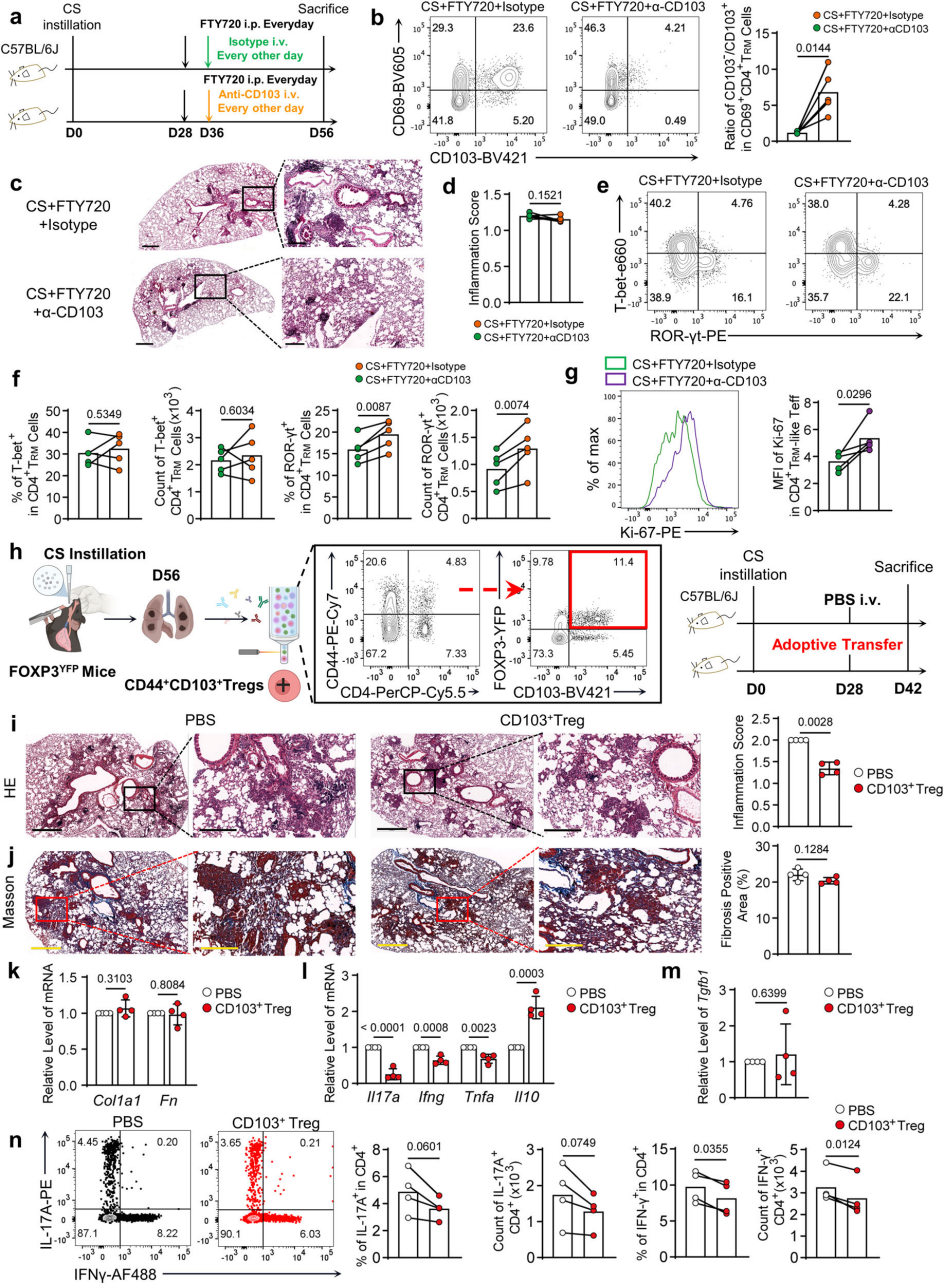
CD103^+^ T_RM_-Tregs exerted immuno-suppressive but not pro-fibrotic roles in the progression of silicosis. **a** Experiment design for CD103 neutralizing: silicotic mice treated with FTY720 (D28– D56 20 μg i.p.) together with CD103 neutralizing or isotype antibodies (100 μg/time i.v.) at indicated time points. **b** FC analysis of CD4^+^ T_RM_ cells for CD69 and CD103 expressions. The bar graph compared the ratios of CD103^–^ to CD103^+^ in the CD69^+^ CD4^+^ T_RM_ cells. **c** and **d** H&E staining to the lung sections of silicotic mice treated with FTY720 together with CD103 neutralizing or isotype antibodies. Scale Bar = 500 μm (left) and 200 μm (right). **e** and **f** FC analysis of the percentages and numbers of T-bet^+^ or ROR-γt^+^ in pulmonary CD4^+^ T_RM_ cells. **g** Flow histogram compared MFI of Ki-67 in the CD4^+^ T_RM_-Teff cells. Bar graph displayed the MFI. **h** The schematic diagram describes the process of CD103^+^ T_RM_-Tregs (CD4^+^ CD44^+^ FOXP3^YFP+^ CD103^+^) sorted in 8 weeks post-CS-treated FOXP3^YFP^ mice by the FACS method. CS-pre-challenged mice were i.v. transferred with the sorted CD103^+^ Tregs or PBS. The recipient mice were analyzed 14 days after cell transfer. **i** H&E staining to the lung sections of distinct groups. Scale Bar = 600 μm (left) and 300 μm (right). The graph shows the inflammation score. **j** Masson’s trichrome staining to the lung sections of different groups. Scale Bar = 600 μm (left) and 300 μm (right). The bar graph showed the fibrosis-positive area. **k** Relative mRNA levels of *Col1a1* and *Fn* in each group. **l**, **m** Relative mRNA levels of *Il17a*, *Ifng*, *Tnfa*, *Il10* (l), and *Tgfb1* (m) in each group. **n** FC analysis of the ratios and counts of IL-17A^+^ and IFN-γ^+^ in CD4^+^ T cells. For panels **a**–**g**, *n* = 4–5 biologically independent animals. For panels **i**–**n**, littermate mice were used, *n* = 4 biologically independent animals. *N* number also indicated independent experimental replicates. Individual mice are plotted on the graphs. The black line indicates the data were analyzed in parallel. Values are reported as the mean ± SD. *P* value was determined by unpaired two-tailed Student’s *t*-test or paired two-tailed Student’s *t*-test.

Next, we aim to explore whether targeting the CD103^+^ T_RM_ subset exerts protective effects on silicosis progression. Taking the notion that CD103^+^ T_RM_-Tregs were preferentially recruited from the circulation, we employed FTY720 plus CD103 neutralization treatments to deplete the T_RM_ subsets (Fig. 5a). Significantly, the treatment diminished CD103^+^ T_RM_ subsets, resulting in a higher CD103^–^/CD103^+^ ratio (Fig. 5b). Unexpectedly, we observed a semblable phenotype of the silicotic lungs, suggesting anti-CD103 treatment did not exert protective roles (Fig. 5c, d). Furthermore, we observed surged ratios of ROR-γt^+^ (Th17-type T_RM_) cells, while T-bet^+^ (Th1-type T_RM_) counterparts were not significantly affected (Fig. 5e, f). Significantly, depleting CD103^+^ T_RM_-Treg augmented Ki-67 levels of the CD103^–^ T_RM_ subsets (most T_RM_-Teff cells) (Fig. 5g), implying depleting CD103^+^ T_RM_ cells unleashed T_RM_-Teff cell proliferation^30^. Collectively, these results demonstrated targeting CD103^+^ T_RM_ cells could not mitigate CS-induced pulmonary fibrosis, which was related to the expansion of T_RM_-Teff cells (CD103^–^ T_RM_).

### CD103^+^ T_RM_-Tregs exerted immuno-suppressive but not pro-fibrotic roles in the progression of silicosis

To study the role of CD103^+^ T_RM_ Tregs in silicosis, we depicted its immunophenotype compared to the CD103^–^ counterparts by flow analysis. We observed higher IL-10 but not TGF-β (detected by LAP, essential for TGF-β cleaved) expressions in the CD103^+^ Tregs (Fig. S2a), demonstrating potent immunosuppressive property but not tissue-repair phenotype^30^. Moreover, there were more effector (PD-1^+^CD25^+^) and terminally differentiated (KLRG1^+^ICOS^+^) phenotypes in CD103^+^ Treg subsets (Fig. S2b, c)^31,32^. Additionally, we observed higher CD39 and CD69 levels in the CD103^+^ Tregs (Fig. S2d), suggesting high immune-suppressive and tissue-resident properties of the CD103^+^ subsets. These results indicated the CD103^+^ Tregs exerted high immune suppressive potentials.

To directly prove the role of CD103^+^ Tregs in the progression of silicosis, we did cell transfer experiments^30^. The CD103^+^ Tregs were sorted from the silicotic lung of FOXP3-YFP mice and transferred into another CS-pre-treated mouse (Fig. 5h). By histological analysis of H&E and Masson trichrome staining to the lung sections, we observed decreased inflammatory leukocyte infiltration (Fig. 5i), but unaffected collagen deposition (Fig. 5j). We further did qPCR analysis to the lung, and confirmed that *Col1a1* and *Fn* transcripts were unaffected (Fig. 5k), indicating that CD103^+^Tregs did not exert profibrotic roles. While the pro-inflammatory mediators *Il17a, Ifng*, and *Tnfa* were descended, the immune suppressive cytokine *Il10* was lifted in the lung tissue transferred with CD103^+^ Tregs (Fig. 5l). In contrast, the cell transfer did not affect *Tgfb1* transcripts, further evidencing that CD103^+^ Tregs did not exert profibrotic roles (Fig. 5m). Furthermore, we detected proinflammatory cytokine productions of CD4^+^ Teff cells. It was demonstrated that appending CD103^+^ Tregs significantly reduced IL-17A and IFN-γ-producing Teff cells in the lung (Fig. 5n), but did not affect IL-13 production (Fig. S2e). Collectively, these results validated that CD103^+^ Tregs functioned as an immunosuppressive regulator restraining Teff cells rather than profibrotic mediators.

### Neutralizing IL-7 in lung retarded silicosis progression through disrupting the pathogenic T_RM_-Teff cell maintenance

**Fig. 6:**
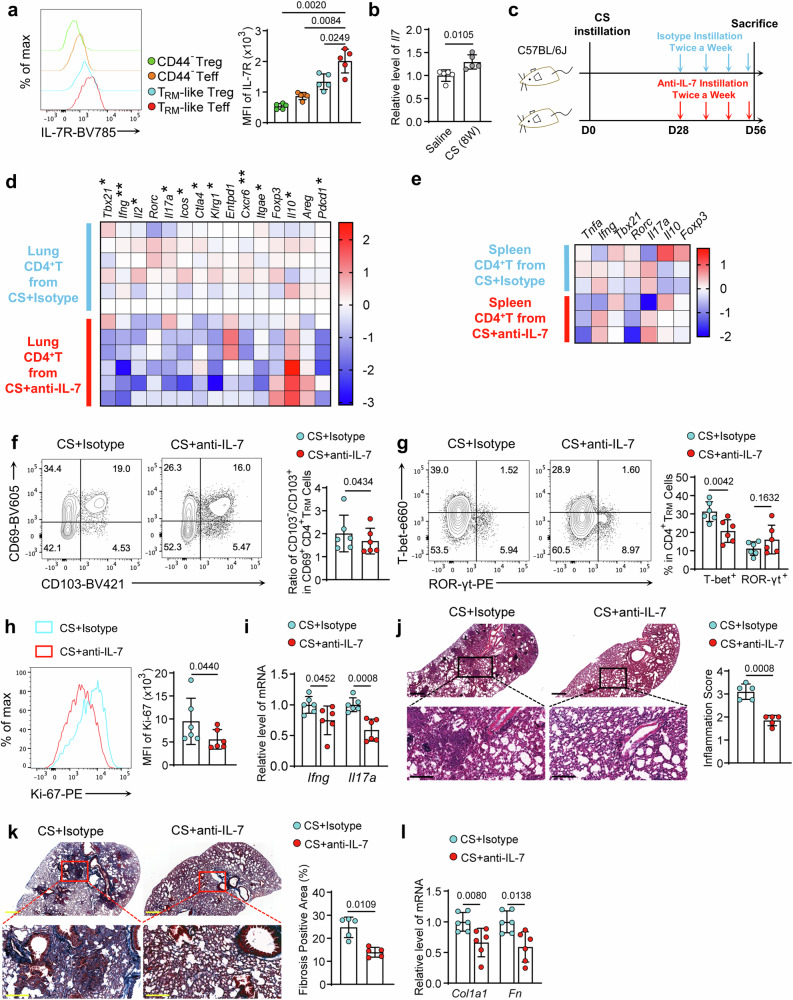
Neutralizing IL-7 in the lung retarded silicosis progression through disrupting the maintenance of pathogenic T_RM_-Teff cells. **a** Flow histogram of IL-7R (CD127) on naive or T_RM_-Tregs and Teffs. The graph compared the MFI. **b** Relative mRNA levels of *Il7a* in mice lung tissue of saline- or CS-treated mice. **c** Bioluminescence of silicotic mice with isotype-control or IL-7 neutralizing (30 µg i.t. twice a week from D28). **d** RNA analysis of sorted pulmonary CD4^+^ T cells in distinct groups. The heatmap contained Th1, Th17, and Treg-related genes, activation, and retention markers. *P < 0.05; **P < 0.01. **e** RNA analysis of sorted splenic CD4^+^ T cells in distinct groups. The heatmap contained Th1, Th17, and Treg-related genes. **f** FC analysis of CD69 and CD103 expression in CD4^+^ T_RM_ cells. The ratios of CD103^–^ to CD103^+^ in the CD69^+^ CD4^+^ T_RM_ cells were shown. **g** FC analysis of CD4^+^ T_RM_ cells for T-bet and ROR-γt expression. The graph displayed the percentages of T-bet^+^ or ROR-γt^+^ T_RM_ cells. **h** Flow histogram indicated the Ki-67 levels in the T_RM_-Teffs. The graph compared the MFI. **i** Relative mRNA levels of *Ifng* and *Il17a* in mice lung tissue of each group. **j** H&E staining to the lung sections of different groups. Scale Bar = 500 μm (upper) and 200 μm (lower). The inflammation scores were measured. **k** Masson’s trichrome staining to the lung sections of different groups. Scale Bar = 500 μm (upper) and 200 μm (lower). The fibrosis-positive areas were quantified. **l** Relative mRNA levels of *Col1a1* and *Fn* in mice lung tissue of each group. For panel **a**, *n* = 5 biologically independent animals. *P* value was determined using one-way ANOVA followed by Tukey’s test. For panels **b**–**l**, *n* = 5–6 biologically independent animals. Individual mice are plotted on the graphs. *N* number also indicated independent experimental replicates. *P* value was determined by unpaired Student’s *t*-test. Values are reported as the mean ± SD.

Now that the CS-induced pathogenic CD4^+^ T_RM_ cells expanded in situ, we next explored interventions targeting their maintenance in the lung, in which IL-7 was reported to be essential^33^. We first examined the IL-7R (CD127) levels on the CD4^+^ T_RM_ cells in silicotic lung. Expectedly, CD4^+^ T_RM_ cells expressed higher levels of IL-7R than the naive T cells (CD44^–^). Strikingly, within CD4^+^ T_RM_ cells, T_RM_-Teff cells, but not T_RM_-Tregs, expressed a higher level of IL-7R, indicating a high demand for IL-7 of those Teff cells (Fig. 6a). Accordingly, the surged level of *Il7* expression in the silicotic lung was confirmed (Fig. 6b). We then aimed to treat the silicotic mice with IL-7-neutralizing antibody. Since IL-7 was critical in lymphocyte maintenance, we chose to treat the mice through intratracheal (i.t.) instillation to avoid affecting the immune response in other organs (Fig. 6c). We sorted CD4^+^ T cells from the lungs and spleens, then did qPCR analysis. Significantly, in the treated pulmonary CD4^+^ T cells, we observed alleviated Th1 and Th17-related transcripts (*Tbx21*, *Ifng*, *Il2*, *Rorc*, and *Il17a*), as well as decreased mRNA levels associated with cell activation (*Icos*, *Ctla4*, *Klrg1*, *and Pdcd1*). Additionally, the cell retention markers (*Cxcr6* and *Itgae*) were decreased, implying a reduction of T_RM_ cells (Fig. 6d). Strikingly, the anti-IL-7 treatment augmented *Il10* transcripts, while the markers related to Tregs, *Foxp3*, and *Areg* were unaltered (Fig. 6d). However, these effects were diminished in the splenic CD4^+^ T cells (Fig. 6e), suggesting that pulmonary local IL-7 neutralization did not affect immune response in whole bodies.

To further detect the effects on CD4^+^ T_RM_ subsets, we did flow analysis and observed a decreased ratio of CD69^+^CD103^–^ to CD69^+^CD103^+^ (Fig. 6f), indicating that the pathogenic CD69^+^CD103^–^ subsets were restrained. By scrutinizing the composition of subsets within CD4^+^ T_RM_ cells, we proved pulmonary IL-7 neutralization blunted T-bet^+^ Th1-type T_RM_, but not the ROR-γt^+^ Th17-type T_RM_ cells (Fig. 6g). In contrast, the unaltered Treg ratio excluded its influences on T_RM_-Tregs, further confirmed by Ki-67 and CD25 levels (Fig. S3a, b). Notably, pulmonary IL-7 neutralization restrained T_RM_-Teff cell proliferation, manifested by decreased Ki-67 levels (Fig. 6h), confirming that IL-7 promoted the expansion of T_RM_-Teff cells^34^. We also did a PCR analysis of the lung tissues. The Th1-related cytokine (*Ifng*), as well as Th17-related cytokine (*Il17a*) transcripts, were decreased in the lung (Fig. 6i), which was in line with previous results. We performed lung histological analysis and validated that IL-7 neutralization reduced immune infiltration, cellular nodule formation (Fig. 6j), and attenuated collagen deposition (Fig. 6k). Further, the declined *Col1a1* and *Fibronectin* transcripts in the cytokine-neutralized lung validated the alleviated fibrosis phenotype (Fig. 6l). Collectively, these data demonstrated that pulmonary local IL-7 intervention restrained CS-induced pulmonary fibrosis by disrupting the T_RM_-Teff maintenance and function, highlighting the important role of T_RM_-Teff in mediating silicosis.

## Discussion

Here we showed that a substantial accumulation of pulmonary CD4^+^ T_RM_ cells mediated the pathogenesis of silicosis, which exerted immunological memory and antigen-specific response to the invaded CS particles. The CD4^+^ T_RM_ cells, especially CD69^+^CD103^+^ T_RM_-Tregs, rely on continual replenishment from circulating lymphocytes. The defined population by CD103 and CD69 displayed distinct functional phenotypes. Targeting the immunosuppressive CD103^+^ T_RM_ cells did not exert protective roles to silicosis. Whereas neutralizing IL-7 in the lung disrupted the maintenance of T_RM_-Teff cells during silicosis progression and exerted protective effects.

Since the invaded CS particles could not be cleared by the pulmonary immune system, it would lead to repeated and cycled antigen stimulation, in which our one-time intratracheal CS instillation gave rise to CD4^+^ T_RM_ cells. *Rag1*^–/–^ mice transfer experiment and repeated CS exposure jointly revealed that new CS exposure induced rapid T_RM_ cell expansion and potent reaction, demonstrating their immunologic memory and antigen-specific characterization. Notably, the CS particles could induce T-cell proliferation by directly activating cells through T-cell antigen receptor (TCR) complexes but not rely on antigen-presenting cells^35^. Additionally, the rearrangement of TCR was confirmed by TCR repertoire sequencing under silica particle exposure^36^. Further study about the structural or molecular mechanism of how CS particles activate T cells, for instance, exploring the CS binding site within the TCR footprint or the presenting MHC molecule and the peptide bound by the MHC, would provide insights into the disease mechanisms and targets for antigen-specific therapies.

By circulatory lymphocyte blockade and parabiosis experiment, we proved the circulating T cells contributed to expanded T_RM_ cells, which confirmed that lymphocytes migrating from lymphoid organs to tissues are the origin of T_RM_ cell formation^12^. Analog to our results, the circulatory T cells contribute to CD4^+^ T_RM_ cells exacerbating asthma^37^. Significantly, we noticed circulating lymphocyte recruitment blockage by FTY720 decreased the percentage of Tregs in CD4^+^ T_RM_, implying their high demand for replenishment from circulating lymphocytes. Ultimately, our parabiosis experiment directly proved CD69^+^CD103^+^ T_RM_-Tregs depended more on circulatory T cell replenishment. In supporting the notion that we previously reported the T_RM_-Tregs in the silicotic lung expressed high Nrp-1 (markers of thymus-derived Tregs)^23,38^, further proving the T_RM_-Tregs originally came from the thymus and recruited to peripheral tissue, but not the conversion of Teff cells to Tregs^39^. All the evidence supported the suggestion that circulating thymus-derived Tregs are required to replenish CD69^+^CD103^+^ T_RM_-Tregs to ensure a tissue-local immunosuppressive environment.

Remarkably, even though FTY720 treatment could attenuate silicosis following our results, it should be cautious. A common adverse effect would be an increased incidence of respiratory infections because of systemic immune cell deficiency^40^. Hence, we further explore the subsets of CD4^+^ T_RM_ cells, which could provide more available interventions. The CS-stimulated pulmonary CD4^+^ T_RM_ cells expressed CD69, regardless of Teff cells or Tregs, implying a crucial role of this molecule in silicosis. However, research reported genetically deleting CD69 in mice did not affect CD4^+^ T_RM_ cells^41^, indicating that CD69 per se is not necessarily sufficient for influencing T_RM_ cell maintenance or function. Analogous to the immuno-suppressive CD69^+^CD103^+^ Tregs in silicosis, CD69^+^ Tregs protect from inflammatory damage after myocardial infarction^42^. Surprisingly, the prominent expression of CD103 was also confirmed on cytotoxic T_RM_ cell subsets, like the CD103^−^ subsets in silicosis^43,44^. We speculated that tissue microenvironment and disease model contributed to the distinction of T_RM_ cell phenotypes^45^. Thus, a comprehensive understanding of the effects of CD69 and CD103 on the function and immuno-balance within CD4^+^ T_RM_ cells still needs dissection.

Despite numerous studies exploring the role of Tregs in fibrotic disease, this topic is still debatable^46,47^, related to Tregs’ heterogeneity in peripheral tissues^48,49^. In pursuing the role of circulatory-derived CD103^+^ T_RM_-Tregs in silicosis, we applied FTY720 together with CD103 neutralizing antibody to the silicotic mice, expecting to get an alleviated effect. Surprisingly, this treatment did not mitigate disease phenotype. While the restrained effector T proliferation was unleashed by CD103 neutralization, consistent with the notion that CD103^+^ T_RM_-Tregs restrained pathogenic CD4^+^ Teff cells in the lung^50^. By direct cell transfer experiment, we demonstrated CD103^+^ T_RM_-Tregs limited proinflammatory cytokine productions and exerted inhibitory effects on chronic inflammation, but could not alleviate fibrosis. Because the replenishment of T_RM_-Tregs was highlighted by our parabiosis experiment, a shortcoming of our study was that one-time cell transfer might not maintain long-lasting effects of CD103^+^ T_RM_-Tregs against fibrogenesis. Furthermore, research about markers distinguishing pro-fibrotic or immunosuppressive Tregs would provide insights into treating silicosis.

Specifically, CD69^+^CD103^–^ T_RM_ possessed a robust proinflammatory response to invaded particles manifested as pathogenic Th1 and Th17-type T_RM_-Teff cells^51^, which was in line with the observation of vaccine or transplantation‐induced Th1-like CD69^+^CD103^–^ T_RM_ subsets^26,52^. The invaded CS particles damaged lung tissue and triggered inflammatory responses, while fibrogenesis was pathological excessive tissue repairment. We found that CD69^+^CD103^–^ CD4^+^ T_RM_ cells expanded in response to repeated CS stimulation, secreting pro-inflammatory mediators IFN-γ and IL-17A^53,54^. Since the particles could not be cleared from lung tissue, repeated CS stimulation would lead to proinflammatory T_RM_ cell expansion and enlarge lung local damage, eventually promoting the progression of silicosis^55^. Thus, modulating immuno-balance within CD4^+^ T_RM_ cells that restrained the number and function of CD69^+^CD103^−^ T_RM_ subsets may provide therapeutic effects in treating silicosis.

IL-7 are crucial cytokines produced by pulmonary fibroblasts and epithelial cells in fibrosis, regulating lymphocyte development and maintaining T_RM_ cell viability^56,57^. Herein, we illuminated T_RM_-Teff cells expressed higher IL-7R, indicating the critical role of IL-7 in cell maintenance and function. Higher cytokine level of IL-7 in the serum was observed in silicosis patients^58^, whose role in silicosis was elusive. Although the suppressive role of IL-7 on TGF-β signaling has been validated^59^, the maintenance of pathogenic T_RM_ subsets via IL-7 could also aggravate tissue injury and subsequent pulmonary fibrosis, by producing other pro-inflammatory cytokines. We demonstrated that IL-7 neutralization in the lung was effective in alleviating CS-induced pulmonary fibrosis by decreasing pathogenic CD4^+^ T_RM_-Teff cells but not T_RM_-Tregs expressing low IL-7R. Neutralizing IL-7 locally in the lung disrupted the maintenance of T_RM_-Teff cells, attenuated CS-induced pulmonary chronic inflammation, mitigated local inflammatory damage to lung tissue, and thus inhibited fibrosis progression (excessive tissue repair). In particular, the Th9 cells might reside in T_RM_-Teff subsets with higher IL-7R expression, which was a major player in inducing fibrosis^60^. Since Th9 cells were involved in IL-7R high T_RM_-Teff subsets, the anti-IL-7 treatment could successfully attenuate the function of Th9 cells, overlapping the effect of anti-IL-9 treatment. Reversely, the successful postponed pulmonary fibrosis under anti-IL-7 blocking might also contain the decreased maintenance of Th9 cells. We did not utilize neutralizing antibodies by intravenous treatment since it may affect immune responses in other organs. Expectedly, lung local IL-7 neutralization did not affect the immune response in the spleen. Besides, bronchoalveolar lavage appending IL-7 neutralizing treatment may provide postponing effects to silicosis in the advanced stage.

In conclusion, our results provided novel functions of CD4^+^ T_RM_ cells in silicosis and new insights into CS particle’s toxicological effects in the lung, all of which will be essential for the development of new therapeutic strategies for postponing the intractable inflammation-associated fibrotic diseases.

## Methods

### Mice

C57BL/6JGpt mice (Strain No. N000013), CD45.1 congenic mice (C57BL/6JGpt-Ptprc^em1Cin(p.K302E)^/Gpt, Strain No. T054816), *Rag1*^–/–^ mice (C57BL/6JGpt-Rag1^em1Cd3259^/Gpt, Strain No. T004753) involved in this study were purchased from GemPharmatech (Nanjing, China). FOXP3^YFP^ mice (B6.129(Cg)-*Foxp3*^tm4(YFP/icre) Ayr^/J, Strain #016959) were purchased from Jackson laboratory. All mice were bred and maintained under specific pathogen-free conditions in the animal care facility at China Medical University. Sex- and age-matched mice were used. All mice were used at the age of 7–8 weeks. Littermate mice were selected if possible. The experimental protocols were approved by the Institutional Animal Care and Use Committees (IACUC) of China Medical University and all animal experiments were performed in accordance with the National Institute of Health Guide for the Care and Use of Laboratory Animals.

### Crystalline silica particles

Crystalline silica (CS) particles were purchased from the U.S. Silica Company (Frederick, MD, USA). The characteristics of crystalline silica particles are described in detail^23^. Briefly, the size distribution of CS particles is as follows: 97% <5 μm in diameter, 80% <3 μm in diameter, median diameter of 1.4 μm. The particles were suspended in sterile saline after drying. The suspension was autoclaved and sonicated for 10 min before use.

### The murine model of silicosis establishment

The mouse model of silicosis was established according to our previously published method^23^. In brief, the mouse was treated with 3.0 mg CS in 50 μL saline solution by intratracheal (i.t.) instillation after anesthetization with pentobarbital sodium (30 mg/kg, i.p. injection, Sigma). An equal amount of sterile saline was applied to the control groups. 56 days post-CS instillation was regarded as the stage of fibrogenesis. Besides, the mouse in the CS repeated exposure group was treated with 1.0 mg CS in 50 μL saline solution per week by i.t. instillation for successive three weeks.

### Single-cell suspension preparation

Isolation of lymphocytes from mouse lungs and spleen was previously described^23^. In short, the lung was minced and digested with a digestion solution containing type I collagenase (2 mg/mL), DNase I (100 U/mL), and complete media (DMEM plus 4% BSA). The suspension was incubated on a rocker at 37 °C. Collagen-digested lungs were dispersed. Red blood cells were lysed using ACK lysis buffer. Leukocytes were enriched from the cell digestion using Percoll gradients (80%/40%) (Cytiva). Spleen cells were prepared by pressing the tissues through 70 μm cell strainers using the end of a sterile plunger of a 5 mL syringe. Single-cell suspension was prepared for subsequent flow cytometry staining or cell sorting.

### Flow cytometry analysis

Flow cytometry (FC) was performed according to the guidance^61^. Live/Dead cell viability dye Aqua (Invitrogen) was used to exclude the influence of dead cells. Fluorescently labeled antibodies to cell surface antigens were applied and incubated at 4 °C for 30 min. For the intracellular transcriptional factor staining, cells were first stained for cell-surface markers and fixed with 4% paraformaldehyde, permeabilized with the FOXP3 transcription factor staining buffer set (eBioscience), and then stained with indicated antibodies to transcriptional factors at 4 °C overnight. For the cytokine staining, the leukocyte stimulation cocktail containing PMA, ionomycin, brefeldin A, and monensin (Invitrogen) was used to stimulate cells for 4 h before staining. The antibodies detecting transcriptional factors or cytokines were incubated at 4 °C overnight. FC analysis was performed on BD FACS Celesta (BD Bioscience, San Jose, CA, USA). FACS data were analyzed with Flowjo 10.6 software. The gating strategy is shown in Fig. S1a. All involved anti-mouse antibodies are listed in Table S1.

### Intravascular immune cell labeling

To discriminate tissue-resident cells from circulating cells, we carried out a well-established intravenous (i.v.) staining approach, illuminated in Fig. 1a^23^. Mice were i.v. injected with 1.5 μg of CD45-APC-Cy7 antibody (30-F11, Biolegend) diluted in 150 μL sterile saline, 4 min before sampling.

### Magnetic-activated cell sorting and adoptive transfer

CD4^+^ αβT cells were purified from the tissues of 8 weeks post CS-treated lung, 8 weeks post CS-treated spleen, and saline-treated spleen by the magnetic-activated cell sorting (MACS) method with a CD4^+^ T cells Isolation Kit (Miltenyi Biotec, Cat#130-104-454). The purity of the isolated cells was >95%. The MACS-purified CD4^+^ T cells or PBS were i.v. transferred into littermate *Rag1*^–/–^ mice (3 × 10^5^ cells per mouse). After that, CS was instilled. One week after the CS treatment, the transferred mice were analyzed. The donor and recipient mice were sex matched.

### Flow cytometric cell sorting and adoptive transfer

FOXP3^YFP+^ mice were treated with CS particles. The activated CD103^+^ Tregs (CD4^+^ CD44^+^ FOXP3^YFP+^ CD103^+^) were sorted from the lung of 8 weeks post-CS-treated FOXP3^YFP+^ mice by flow cytometric cell sorting (FACS Aria II instrument, BD Biosciences). The gating strategy is shown in Fig. 5h. The sorted Tregs (1 × 10^4^ cells each mouse) or PBS were i.v. transferred into littermate C57BL/6J mice treated with CS 4 weeks before transfer. Two weeks later, the reconstituted mice were analyzed. The donor and recipient mice were sex matched.

### Parabiosis model

The experiment was adopted as the published protocols^26,27^. Briefly, sex- and age-matched congenic mice (CD45.1/2 and CD45.1/1) were co-caged and prepared. Their dorsal and ventral skin were approximated with sutures after parabiosis surgery. The conjoined mice recovered for 14 days to establish new circulation. Then, CD45.1/1 mice were CS administered, and sacrificed 7 days later for flow analysis.

### RNA extraction and quantitative PCR (qPCR) analysis

RNA was extracted from lung tissue by RNA isolater Total RNA Extraction Reagent (R401, Vazyme Biotech), then reversely transcribed into cDNA by HisScript IIIs RT SuperMix (R323, Vazyme Biotech). The ChamQ Universal SYBR qPCR Master Mix (Q711, Vazyme Biotech) was used for the amplification of RNA samples from each group and gene expression was analyzed via real-time PCR assay (7500 software, Applied-biosystem). GAPDH was used as the internal control for determining 2^−ΔΔCT^ values. All primers were synthesized by Sangon Biotech, and the sequences are listed in Table S2.

### FTY720 treatment

FTY720, an agonist of sphingosine-1-phosphate receptor 1 (S1PR1), was used to block lymphoid cell migration. FTY720 (20 μg in the vehicle every time) or vehicle was *i.p*. administered daily (Fig. 3a). Littermate mice were used in each independent experiment. The mice were divided into three groups: (A) CS-treated group, in which animals received CS instillation and vehicle; (B) Full-time FTY720 treatment (0–8 weeks), in which the mice received CS instillation combined with FTY720 i.p. every day from 0 to 8 weeks; (C) Half-time FTY720 treatment (4–8 weeks), in which the mice received CS instillation combined with FTY720 i.p. every day from 4 to 8 weeks.

### Neutralizing antibody treatments

Anti-CD103 treatment: Anti-mouse CD103 (M290, BE0026) and isotype-matched antibodies were purchased from Bio X cell. C57BL/6J mice were divided into two groups (Fig. 5a): Isotype group, in which mice received CS instillation and FTY720 treatment (D28 to D56 every day) together with isotype antibody (100 µg i.v. D36– D56 every other day). Anti-mouse CD103 group, in which the mice received CS instillation and FTY720 treatment (D28 to D56 every day) and anti-mouse CD103 antibody (100 µg i.v. D36 to D56 every other day). Anti-IL-7 treatments: Anti-mouse IL-7 antibody (M25, BE0048) and isotype-matched antibody were purchased from Bio X cell. Briefly, C57BL/6J mice were divided into an anti-IL-7 group and an Isotype group, in which mice received CS instillation and isotype or anti-mouse IL-7 antibody (30 µg, i.t., twice a week from D28 to D56).

### Lung histological analysis

The lung tissues of mice were fixed with 4% paraformaldehyde (PFA) and embedded in paraffin. Slices (4 μm thick) were cut, mounted on slides, and stained. Hematoxylin & Eosin (H&E) were conducted to assess the degree of inflammation and pathological changes. Collagen fiber content was quantified using Masson’s trichrome staining. The staining was performed according to the manufacturer’s protocol. Stained lung sections were photographed in a microscope (Leica M205 FA, Wetzlar, Germany). The inflammation score was done by a semi-quantitative analysis based on a previously published method^23^. Briefly, lung inflammation was graded into four stages and scored as follows: normal lung, 1 point; light inflammation, 20% of lung area, 2 points; medium inflammation, 20–50% of lung area, 3 points; severe inflammation, 50% of lung area, severe structure distortion, 4 points. The fibrotic area is presented as a percentage, measured by Image J.

### Statistics and reproducibility

It was assumed that sampling was from a normally distributed population. Statistical analysis was performed using GraphPad Prism 9.0.1 software. Unpaired/paired two-sided Student’s *t*-test (2 groups) or one-way analysis of variance (ANOVA) followed by Tukey’s test (more than 2 groups) was used to evaluate the statistical differences between groups. All data were presented as the Mean ± SD for bar graphs unless otherwise stated, with sample size (*n*) specified in figure legends. Absolute *P* values are indicated in each figure. For all analyses, a *P* value < 0.05 was considered to indicate significance. Experimental results were reliably replicated. For each experiment, the number of replications was noted in figure legends.

### Study approval

The experimental protocols were approved by the Institutional Animal Care and Use Committees (IACUC) of China Medical University, and we have complied with all relevant ethical regulations for animal use, in accordance with the National Institute of Health Guide for the Care and Use of Laboratory Animals.

### Reporting summary

Further information on research design is available in the Nature Portfolio Reporting Summary linked to this article.

### Supplementary information

Supplementary Information

Description of additional supplementary files

Supplementary Data 1

Reporting Summary

## Data Availability

The source data underlying the graphs of this study can be found in Supplementary Data 1.
