# Supplementary material for: Crystalline silica-induced recruitment and immuno-imbalance of CD4+ tissue resident memory T cells promote silicosis progression
Source: Commun Biol. 2024 Aug 9;7:971. doi: 10.1038/s42003-024-06662-z (PMC11316055; doi:10.1038/s42003-024-06662-z)
Supplement: Supplementary file 1 — Supplementary Information [file 42003_2024_6662_MOESM1_ESM.pdf]

Supplemental Information

Crystalline silica-induced recruitment and immuno-imbalance of CD4$^+$ Tissue Resident Memory T cells promote silicosis progression.

Yichuan You[1, 2#], Xiulin Wu[1, 2#], Haoyang Yuan[1, 2], Yangyang He[1, 2], Yinghui Chen[1, 2], Sisi Wang[1, 2], Hui Min[3], Jie Chen[1, 2*], Chao Li[1, 2, 4*].

1 Key Laboratory of Environmental Stress and Chronic Disease Control & Prevention (China Medical University), Ministry of Education, No.77 Puhe Road, Shenyang North New Area, Shenyang, 110122, Liaoning, PR China

2 Department of Occupational and Environmental Health, School of Public Health, China Medical University, No.77 Puhe Road, Shenyang North New Area, Shenyang, 110122, Liaoning, PR China

3 Department of Immunology, College of Basic Medical Sciences, China Medical University, No.77 Puhe Road, Shenyang North New Area, Shenyang, 110122, Liaoning, PR China

4 Lead contact.

# These authors contribute equally to this work.

* Corresponding author.

* Correspondence: lichao@cmu.edu.cn (C. Li); jchen@cmu.edu.cn (J. Chen)

Supplementary Information

Supplementary Figures

Supplementary Figure 1: CS particles stimulated CD4^{+} T_{RM} cell emergence.

Supplementary Figure 2: CD103^{+} T_{RM}-Tregs exerted immuno-suppressive without pro-fibrotic roles.

Supplementary Figure 3: Neutralizing IL-7 in the lung affected the activation of repressive T_{RM}-Tregs.

Supplementary Tables

Supplementary Table 1. List of antibodies used for flow cytometry

Supplementary Table 2. Primer sequences for qPCR in this study

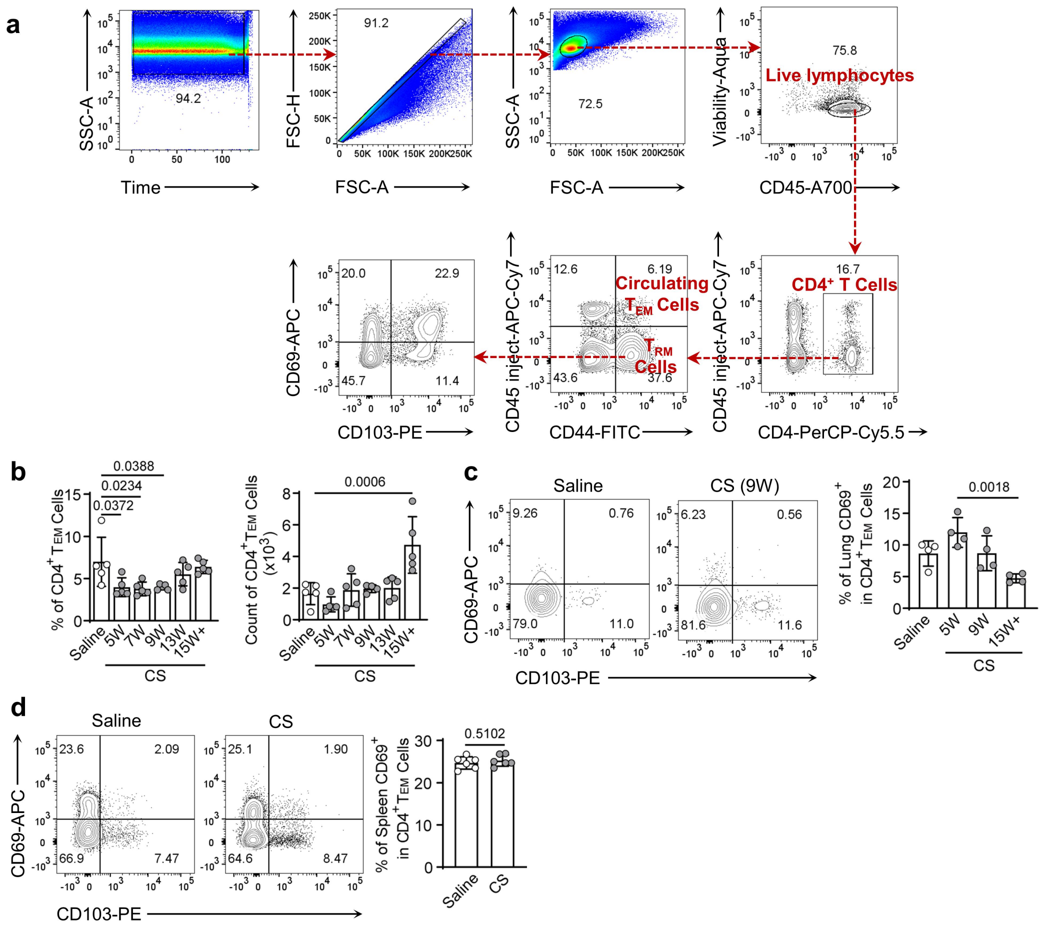

Figure S1. CS particles stimulated CD4⁺ T_{RM} cell emergence. (a) Gating strategy of flow cytometry analysis. (b) Percentages and counts of the CD4⁺ T_{EM} cells were compared at the indicated time points (n = 5). (c) Flow cytometry (FC) analysis of lung circulating T_{EM} cells for CD69 and CD103 expression of saline or CS-treated mice at specified time points. The bar graph illustrated ratios of CD69⁺ in circulating CD4⁺ T_{EM} cells (n = 4). (d) FC analysis of splenic T_{EM} cells for CD69 and CD103. The graph compared percentages of CD69⁺ on splenic CD4⁺ T_{EM} cells of saline or CS-treated mice (n = 6). The bar graphs are the combined results of at least three independent experiments. Individual mice are plotted on the graphs. Values are reported as the mean ± SD. *P* value was determined by one-way ANOVA followed by Tukey's test or unpaired two-tailed Student's *t*-test.

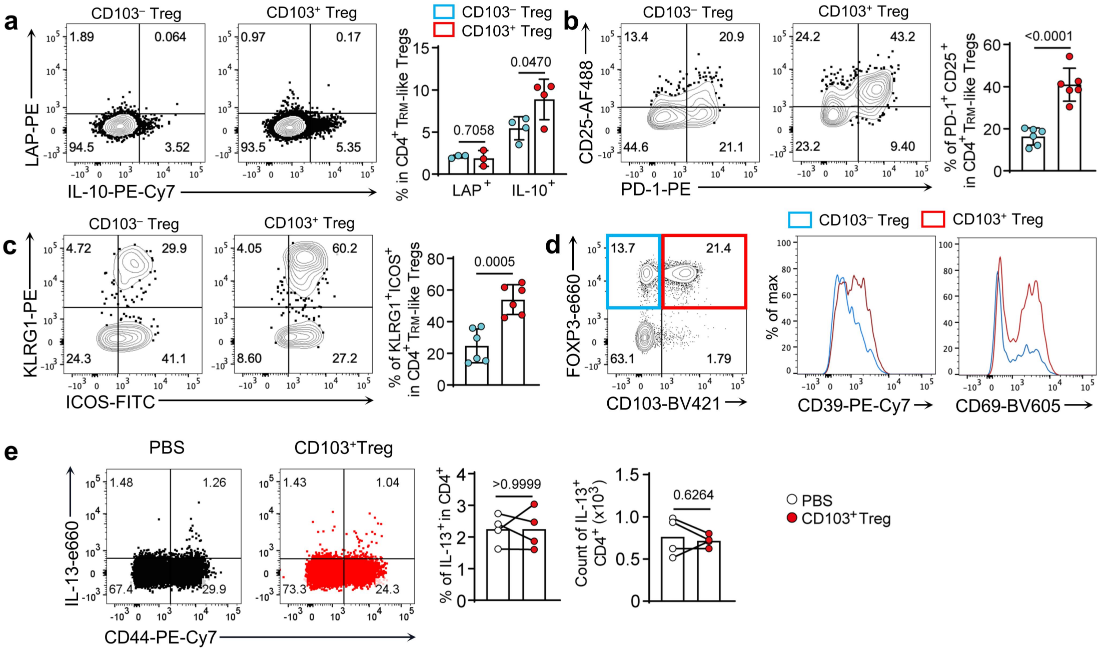

Figure S2. CD103⁺ TRM-Tregs exerted immuno-suppressive without pro-fibrotic roles. (a) FC analysis of CD103⁺ and CD103⁻ Tregs for IL-10 and LAP expressions. The graph compared the percentages of IL-10⁺ and LAP⁺ (n = 3 to 4). (b) FC analysis of effector phenotype (CD25⁺PD-1⁺) within CD103⁺ and CD103⁻ Tregs. The graph compared the proportion (n = 6). (c) FC analysis of terminal differentiated phenotype (ICOS⁺KLRG1⁺) within CD103⁺ and CD103⁻ Tregs. The graph compared the ratios (n = 6). (d) Flow histogram compared the expression of CD39 and CD69. (e) FC analysis of IL-13⁺ in CD4⁺ T cells. The graph compared the ratios and counts of IL-13⁺ in CD4⁺ T cells (n = 4). Individual mice are plotted on the graphs. Values are reported as the mean ± SD. *P* value was determined using unpaired or paired two-tailed Student's *t*-test.

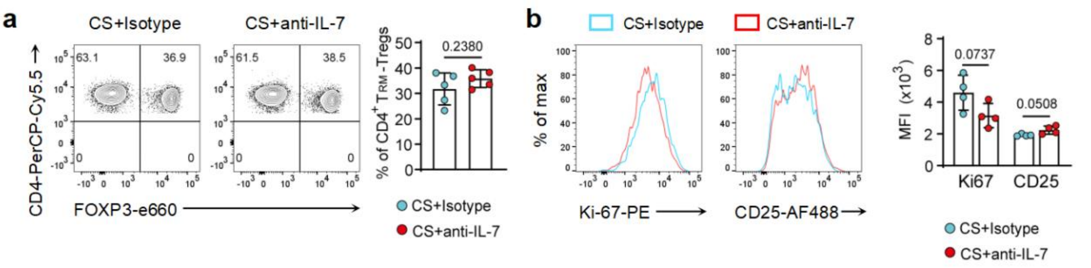

Figure S3. Neutralizing IL-7 in the lung affected the activation of repressive T_{RM}-Tregs.

(a) FC analysis of FOXP3⁺ Tregs in T_{RM} cells after anti-IL-7 treatment. The graph compared the percentages of FOXP3⁺ (n = 5). (b) Typical flow histogram compared the expression of Ki-67 and CD25. The graph compared the MFI (n = 4). Individual mice are plotted on the graphs. Values are reported as the mean ± SD. *P* value was determined using unpaired two-tailed Student's *t*-test.

Supplementary Tables

Table S1. List of antibodies used for flow cytometry

Target	Conjugate	Clone	Vendor	Identifier
**CD45**	APC-Cy7	30-F11	Biolegend	Cat#103116<br>RRID: AB_312981
**CD45**	A700	30-F11	Biolegend	Cat#103127<br>RRID: AB_493714
**CD44**	PE-Cy7	IM7	Biolegend	Cat#103030<br>RRID: AB_830787
**CD39**	PE-Cy7	Duha59	Biolegend	Cat#143805<br>RRID: AB_2563393
**streptavidin**	PE-Cy7		Biolegend	Cat#405206
**IL-10**	biotin	JES5-16E3	Biolegend	Cat#505003<br>RRID: AB_315357
**CD103**	BV421	2E7	Biolegend	Cat#121422<br>RRID: AB_2562901
**CD69**	BV605	H1.2F3	Biolegend	Cat#104530<br>RRID: AB_2563062
**CD4**	PerCP-Cy5.5	GK1.5	Biolegend	Cat#100434<br>RRID: AB_893324
**CD25**	AF488	PC61.5	eBioscience	Cat#53-0251-82<br>RRID: AB_763472

**ICOS**	FITC	C398.4A	Biolegend	Cat#313505<br>RRID: AB_416329
**CD44**	FITC	IM7	Biolegend	Cat#103006<br>RRID: AB_312957
**LAP-TGF-β1**	PE	TW7-16B4	Biolegend	Cat#141403<br>RRID:AB_10730610
**KLRG1**	PE	2F1/KLRG1	Biolegend	Cat#138408<br>RRID:AB_10574313
**PD-1**	PE	29F.1A12	Biolegend	Cat#135205<br>RRID: AB_1877232
**Ki-67**	PE	16A8	Biolegend	Cat#652404<br>RRID: AB_2561525
**ROR-γt**	PE	B2D	eBioscience	Cat#12-6981-80<br>RRID:AB_10805392
**T-bet**	e660	eBio4B10 (4B10)	eBioscience	Cat#50-5825-80<br>RRID:AB_10597459
**GATA3**	e660	TWAJ	eBioscience	Cat#50-9966-42<br>RRID:AB_10596663
**FOXP3**	e660	FJK-16s	eBioscience	Cat#50-5773-82<br>RRID:AB_11218868
**Viability dye**	Aqua	Dead cell	Invitrogen	Cat#L34966
**CD69**	APC	H1.2F3	Biolegend	Cat#104514

				RRID:AB_492843
**CD103**	PE	2E7	eBioscience	Cat#12-1031-82<br>RRID:AB_465799
**CXCR6**	BV421	SA051D1	Biolegend	Cat#151109<br>RRID:AB_2616760
**PD-1**	BV421	29F.1A12	Biolegend	Cat#135217<br>RRID:AB_10900085
**ST2**	BV421	U29-93	BD Biosciences	Cat#566309<br>RRID:AB_2744489
**IL-17A**	PE	TC11-18H10.1	Biolegend	Cat#506904<br>RRID:AB_315464
**IFN-γ**	AF488	XMG1.2	BD Biosciences	Cat#557724<br>RRID:AB_396832
**IL-13**	e660	eBio13A	eBioscience	Cat#50-7133-82<br>RRID:AB_2574279
**CD45.1**	BV510	A20	Biolegend	Cat#110741<br>RRID:AB_2563378
**CD45.2**	A700	104	Biolegend	Cat#109822<br>RRID:AB_493731
**FOXP3**	PE	FJK-16s	eBioscience	Cat#12-5773-82<br>RRID:AB_465936

Table S2. Primer sequences for qPCR in this study

Gene	Forward (5'-3')	Reverse (5'-3')
Gapdh	AGGTCGGTGTGAACGGATTTG	TGTAGACCATGTAGTTGAGGTCA
Il17a	TTTAACTCCCTTGGCGCAAAA	CTTTCCCTCCGCATTGACAC
Tbx21	AGCAAGGACGGCGAATGTT	GGGTGGACATATAAGCGGTTC
Tgfb1	CTCCCGTGGCTTCTAGTGC	GCCTTAGTTTGGACAGGATCTG
Rorc	GACCCACACCTCACAAATTGA	AGTAGGCCACATTACACTGCT
Il10	GCTCTTACTGACTGGCATGAG	CGCAGCTCTAGGAGCATGTG
Il2	TGAGCAGGATGGAGAATTACAGG	GTCCAAGTTCATCTTCTAGGCAC
Col1a1	CTGTAACATGGAAACTGGGGAAA	CCATAGCTGAACTGAAAACCACC
Fn	GACCAGTGCCAAGATTCAGAGACC	TTCCTTCCAGCGACCCGTAGAG
Icos	ATGAAGCCGTACTTCTGCCAT	CGCATTTTTAACTGCTGGACAG
Ctla4	TTTTGTAGCCCTGCTCACTCT	CTGAAGGTTGGGTCACCTGTA
Klrg1	GAGCGTAGCTTCCGGGACTA	TGTAAGGAGATGTGAGCCTTTGT
Entpd1	AAGGTGAAGAGATTTTGCTCCAA	TTTGTTCTGGGTCAGTCCCAC
Cxcr6	GAGTCAGCTCTGTACGATGGG	TCCTTGAACTTTAGGAAGCGTTT
Itgae	CCTGTGCAGCATGTAAAAGAATG	CAAGGATCGGCAGTTCAGATAC

Foxp3	CCCATCCCCAGGAGTCTTG	ACCATGACTAGGGGCACTGTA
Tnfa	CCCTCACACTCAGATCATCTTCT	GCTACGACGTGGGCTACAG
Areg	GGTCTTAGGCTCAGGCCATTA	CGCTTATGGTGGAAACCTCTC
Ifng	ATGAACGCTACACACTGCATC	CCATCCTTTTGCCAGTTCCTC
Il6	TAGTCCTTCCTACCCCAATTTCC	TTGGTCCTTAGCCACTCCTTC
Pdcd1	ACCCTGGTCATTCACTTGGG	CATTTGCTCCCTCTGACACTG
