# Supplementary material for: Crystalline silica-induced recruitment and immuno-imbalance of CD4+ tissue resident memory T cells promote silicosis progression
Source: Commun Biol. 2024 Aug 9;7:971. doi: 10.1038/s42003-024-06662-z (PMC11316055; doi:10.1038/s42003-024-06662-z)
Supplement: Supplementary file 4 — Reporting Summary [file 42003_2024_6662_MOESM4_ESM.pdf]

Corresponding author(s): Chao Li

Last updated by author(s): Jun 10, 2024

Reporting Summary

Nature Portfolio wishes to improve the reproducibility of the work that we publish. This form provides structure for consistency and transparency in reporting. For further information on Nature Portfolio policies, see our Editorial Policies and the Editorial Policy Checklist.

Statistics

For all statistical analyses, confirm that the following items are present in the figure legend, table legend, main text, or Methods section.

n/a	Confirmed	
☐	☒	The exact sample size (n) for each experimental group/condition, given as a discrete number and unit of measurement
☐	☒	A statement on whether measurements were taken from distinct samples or whether the same sample was measured repeatedly
☐	☒	The statistical test(s) used AND whether they are one- or two-sided<br>*Only common tests should be described solely by name; describe more complex techniques in the Methods section.*
☒	☐	A description of all covariates tested
☐	☒	A description of any assumptions or corrections, such as tests of normality and adjustment for multiple comparisons
☐	☒	A full description of the statistical parameters including central tendency (e.g. means) or other basic estimates (e.g. regression coefficient) AND variation (e.g. standard deviation) or associated estimates of uncertainty (e.g. confidence intervals)
☐	☒	For null hypothesis testing, the test statistic (e.g. F, t, r) with confidence intervals, effect sizes, degrees of freedom and P value noted<br>*Give P values as exact values whenever suitable.*
☒	☐	For Bayesian analysis, information on the choice of priors and Markov chain Monte Carlo settings
☒	☐	For hierarchical and complex designs, identification of the appropriate level for tests and full reporting of outcomes
☒	☐	Estimates of effect sizes (e.g. Cohen's d, Pearson's r), indicating how they were calculated

Our web collection on statistics for biologists contains articles on many of the points above.

Software and code

Policy information about availability of computer code

Data collection	Flow Cytometry: BD FACS Celesta (BD Biosciences); Cell Sorting: BD FACSAria II (BD Biosciences); Imaging Flow Cytometry: Flowjo 10.6 (BD Biosciences)
Data analysis	- Flow Cytometry data were analyzed using Flowjo 10.6 (BD Biosciences) and Prism 9.0.1 (GraphPad).<br>- Images were analyzed using ImageJ 1.51m9 and Prism 9.0.1 (GraphPad). Lung inflammation was graded into four stages and scored as follows: normal lung, 1 point; light inflammation, 20% of lung area, 2 points; medium inflammation, 20 to 50% of lung area, 3 points; severe inflammation, 50% of lung area, severe structure distortion, 4 points. The fibrotic area is presented as a percentage, which were measured by Image J.<br>- For qPCR analysis, GAPDH was used as the internal control for determining 2-ΔΔCT values.

For manuscripts utilizing custom algorithms or software that are central to the research but not yet described in published literature, software must be made available to editors and reviewers. We strongly encourage code deposition in a community repository (e.g. GitHub). See the Nature Portfolio guidelines for submitting code & software for further information.

Data

Policy information about availability of data

All manuscripts must include a data availability statement. This statement should provide the following information, where applicable:

- Accession codes, unique identifiers, or web links for publicly available datasets
- A description of any restrictions on data availability
- For clinical datasets or third party data, please ensure that the statement adheres to our policy

All the source data underlying the graphs in this study can be found in the Supplementary Data 1.

Research involving human participants, their data, or biological material

Policy information about studies with human participants or human data. See also policy information about sex, gender (identity/presentation), and sexual orientation and race, ethnicity and racism.

Reporting on sex and gender	*Use the terms sex (biological attribute) and gender (shaped by social and cultural circumstances) carefully in order to avoid confusing both terms. Indicate if findings apply to only one sex or gender; describe whether sex and gender were considered in study design; whether sex and/or gender was determined based on self-reporting or assigned and methods used. Provide in the source data disaggregated sex and gender data, where this information has been collected, and if consent has been obtained for sharing of individual-level data; provide overall numbers in this Reporting Summary. Please state if this information has not been collected. Report sex- and gender-based analyses where performed, justify reasons for lack of sex- and gender-based analysis.*
Reporting on race, ethnicity, or other socially relevant groupings	*Please specify the socially constructed or socially relevant categorization variable(s) used in your manuscript and explain why they were used. Please note that such variables should not be used as proxies for other socially constructed/relevant variables (for example, race or ethnicity should not be used as a proxy for socioeconomic status). Provide clear definitions of the relevant terms used, how they were provided (by the participants/respondents, the researchers, or third parties), and the method(s) used to classify people into the different categories (e.g. self-report, census or administrative data, social media data, etc.) Please provide details about how you controlled for confounding variables in your analyses.*
Population characteristics	*Describe the covariate-relevant population characteristics of the human research participants (e.g. age, genotypic information, past and current diagnosis and treatment categories). If you filled out the behavioural & social sciences study design questions and have nothing to add here, write "See above."*
Recruitment	*Describe how participants were recruited. Outline any potential self-selection bias or other biases that may be present and how these are likely to impact results.*
Ethics oversight	*Identify the organization(s) that approved the study protocol.*

Note that full information on the approval of the study protocol must also be provided in the manuscript.

Field-specific reporting

Please select the one below that is the best fit for your research. If you are not sure, read the appropriate sections before making your selection.

☒ Life sciences ☐ Behavioural & social sciences ☐ Ecological, evolutionary & environmental sciences

For a reference copy of the document with all sections, see nature.com/documents/nr-reporting-summary-flat.pdf

Life sciences study design

All studies must disclose on these points even when the disclosure is negative.

Sample size	- In vivo experiments: no statistical method was used to determine sample size. The sample size was increased as the experiments progression to enhance statistical reliability.
Data exclusions	No experimental data were excluded.
Replication	Experimental results were reliably replicated. For each experiment, the number of replications were noted in the figure legends.
Randomization	Littermate mice were grouped according to sex and genotype, which were not randomized.
Blinding	As genotypes and sex of littermate mice were known before experiments, their was no blinding.

Behavioural & social sciences study design

All studies must disclose on these points even when the disclosure is negative.

Study description	*Briefly describe the study type including whether data are quantitative, qualitative, or mixed-methods (e.g. qualitative cross-sectional, quantitative experimental, mixed-methods case study).*
Research sample	*State the research sample (e.g. Harvard university undergraduates, villagers in rural India) and provide relevant demographic information (e.g. age, sex) and indicate whether the sample is representative. Provide a rationale for the study sample chosen. For studies involving existing datasets, please describe the dataset and source.*
Sampling strategy	*Describe the sampling procedure (e.g. random, snowball, stratified, convenience). Describe the statistical methods that were used to predetermine sample size OR if no sample-size calculation was performed, describe how sample sizes were chosen and provide a rationale for why these sample sizes are sufficient. For qualitative data, please indicate whether data saturation was considered, and what criteria were used to decide that no further sampling was needed.*
Data collection	*Provide details about the data collection procedure, including the instruments or devices used to record the data (e.g. pen and paper, computer, eye tracker, video or audio equipment) whether anyone was present besides the participant(s) and the researcher, and whether the researcher was blind to experimental condition and/or the study hypothesis during data collection.*
Timing	*Indicate the start and stop dates of data collection. If there is a gap between collection periods, state the dates for each sample cohort.*
Data exclusions	*If no data were excluded from the analyses, state so OR if data were excluded, provide the exact number of exclusions and the rationale behind them, indicating whether exclusion criteria were pre-established.*
Non-participation	*State how many participants dropped out/declined participation and the reason(s) given OR provide response rate OR state that no participants dropped out/declined participation.*
Randomization	*If participants were not allocated into experimental groups, state so OR describe how participants were allocated to groups, and if allocation was not random, describe how covariates were controlled.*

Ecological, evolutionary & environmental sciences study design

All studies must disclose on these points even when the disclosure is negative.

Study description	*Briefly describe the study. For quantitative data include treatment factors and interactions, design structure (e.g. factorial, nested, hierarchical), nature and number of experimental units and replicates.*
Research sample	*Describe the research sample (e.g. a group of tagged Passer domesticus, all Stenocereus thurberi within Organ Pipe Cactus National Monument), and provide a rationale for the sample choice. When relevant, describe the organism taxa, source, sex, age range and any manipulations. State what population the sample is meant to represent when applicable. For studies involving existing datasets, describe the data and its source.*
Sampling strategy	*Note the sampling procedure. Describe the statistical methods that were used to predetermine sample size OR if no sample-size calculation was performed, describe how sample sizes were chosen and provide a rationale for why these sample sizes are sufficient.*
Data collection	*Describe the data collection procedure, including who recorded the data and how.*
Timing and spatial scale	*Indicate the start and stop dates of data collection, noting the frequency and periodicity of sampling and providing a rationale for these choices. If there is a gap between collection periods, state the dates for each sample cohort. Specify the spatial scale from which the data are taken*
Data exclusions	*If no data were excluded from the analyses, state so OR if data were excluded, describe the exclusions and the rationale behind them, indicating whether exclusion criteria were pre-established.*
Reproducibility	*Describe the measures taken to verify the reproducibility of experimental findings. For each experiment, note whether any attempts to repeat the experiment failed OR state that all attempts to repeat the experiment were successful.*
Randomization	*Describe how samples/organisms/participants were allocated into groups. If allocation was not random, describe how covariates were controlled. If this is not relevant to your study, explain why.*
Blinding	*Describe the extent of blinding used during data acquisition and analysis. If blinding was not possible, describe why OR explain why blinding was not relevant to your study.*

Did the study involve field work? ☐ Yes ☐ No

Field work, collection and transport

Field conditions	*Describe the study conditions for field work, providing relevant parameters (e.g. temperature, rainfall).*
Location	*State the location of the sampling or experiment, providing relevant parameters (e.g. latitude and longitude, elevation, water depth).*
Access & import/export	*Describe the efforts you have made to access habitats and to collect and import/export your samples in a responsible manner and in compliance with local, national and international laws, noting any permits that were obtained (give the name of the issuing authority, the date of issue, and any identifying information).*
Disturbance	*Describe any disturbance caused by the study and how it was minimized.*

Reporting for specific materials, systems and methods

We require information from authors about some types of materials, experimental systems and methods used in many studies. Here, indicate whether each material, system or method listed is relevant to your study. If you are not sure if a list item applies to your research, read the appropriate section before selecting a response.

Materials & experimental systems

n/a	Involved in the study	
☐	☒	Antibodies
☒	☐	Eukaryotic cell lines
☒	☐	Palaeontology and archaeology
☐	☒	Animals and other organisms
☒	☐	Clinical data
☒	☐	Dual use research of concern
☒	☐	Plants

Methods

n/a	Involved in the study	
☒	☐	ChIP-seq
☐	☒	Flow cytometry
☒	☐	MRI-based neuroimaging

Antibodies

Antibodies used

Anti-mouse CD45 APC-Cy7 Biolegend Clone:30-F11; Cat#103116; RRID: AB_312981
Anti-mouse CD45 A700 Biolegend Clone:30-F11; Cat#103127; RRID: AB_493714
Anti-mouse CD44 PE-Cy7 Biolegend Clone:IM7; Cat#103030; RRID: AB_830787
Anti-mouse CD39 PE-Cy7 Biolegend Clone:Duha59; Cat#143805; RRID: AB_2563393
Streptavidin PE-Cy7 Biolegend Cat#405206
Anti-mouse IL-10 biotin Biolegend Clone:JES5-16E3; Cat#505003; RRID: AB_315357
Anti-mouse CD103 BV421 Biolegend Clone:2E7; Cat#121422; RRID: AB_2562901
Anti-mouse CD69 BV605 Biolegend Clone:H1.2F3; Cat#104530; RRID: AB_2563062
Anti-mouse CD4 PerCP-Cy5.5 Biolegend Clone:GK1.5; Cat#100434; RRID: AB_893324
Anti-mouse CD25 AF488 eBioscience Clone:PC61.5; Cat#53-0251-82; RRID: AB_763472
Anti-mouse ICOS FITC Biolegend Clone:C398.4A; Cat#313505; RRID: AB_416329
Anti-mouse CD44 FITCBiolegend Clone:IM7; Cat#103006; RRID: AB_312957
Anti-mouse LAP-TGF-β1 PE Biolegend Clone:TW7-16B4; Cat#141403; RRID:AB_10730610
Anti-mouse KLRG1 PE Biolegend Clone:2F1/KLRG1; Cat#138408; RRID:AB_10574313
Anti-mouse PD-1 PE Biolegend Clone:29F.1A12; Cat#135205; RRID: AB_1877232
Anti-mouse Ki-67 PE Biolegend Clone:16A8; Cat#652404; RRID: AB_2561525
Anti-mouse ROR-γt PE eBioscience Clone:B2D; Cat#12-6981-80; RRID:AB_10805392
Anti-mouse T-bet e660 eBioscience Clone:eBio4B10 (4B10); Cat#50-5825-80; RRID:AB_10597459
Anti-mouse GATA-3 e660 eBioscience Clone:TWAJ; Cat#50-9966-42; RRID:AB_10596663
Anti-mouse FOXP3 e660 eBioscience Clone:FJK-16s; Cat#50-5773-82; RRID:AB_11218868
Anti-mouse CD69 APC Biolegend Clone:H1.2F3; Cat#104514; RRID:AB_492843
Anti-mouse CD103 PE eBioscience Clone:2E7; Cat#12-1031-82; RRID:AB_465799
Anti-mouse CXCR6 BV421 Biolegend Clone:SA051D1; Cat#151109; RRID:AB_2616760
Anti-mouse PD-1 BV421 Biolegend Clone:29F.1A12; Cat#135217; RRID:AB_10900085
Anti-mouse ST2 BV421 BD Biosciences Clone:U29-93; Cat#566309; RRID:AB_2744489
Anti-mouse IL-17A PE Biolegend Clone:TC11-18H10.1; Cat#506904; RRID:AB_315464
Anti-mouse IFN-γ AF488 BD Biosciences Clone:XMG1.2; Cat#557724; RRID:AB_396832
Anti-mouse IL-13 e660 eBioscience Clone:eBio13A; Cat#50-7133-82; RRID:AB_2574279
Anti-mouse CD45.1 BV510 Biolegend Clone:A20; Cat#110741; RRID:AB_2563378
Anti-mouse CD45.2 A700 Biolegend Clone:104; Cat#109822; RRID:AB_493731
Anti-mouse FOXP3 PE eBioscience Clone:FJK-16s; Cat#12-5773-82; RRID:AB_465936
Anti-mouse CD103 Bio X cell Clone:M290; Cat#BE0026
Anti-mouse IL-7 Bio X cell Clone:M25; Cat#BE0048

Validation	All antibodies were commercial. Specificity and validation were provided by manufacturer's technical datasheets and confirmed in Materials and Methods. No further validation was performed.

Eukaryotic cell lines

Policy information about cell lines and Sex and Gender in Research

Cell line source(s)	*State the source of each cell line used and the sex of all primary cell lines and cells derived from human participants or vertebrate models.*
Authentication	*Describe the authentication procedures for each cell line used OR declare that none of the cell lines used were authenticated.*
Mycoplasma contamination	*Confirm that all cell lines tested negative for mycoplasma contamination OR describe the results of the testing for mycoplasma contamination OR declare that the cell lines were not tested for mycoplasma contamination.*
Commonly misidentified lines (See ICLAC register)	*Name any commonly misidentified cell lines used in the study and provide a rationale for their use.*

Palaeontology and Archaeology

Specimen provenance	*Provide provenance information for specimens and describe permits that were obtained for the work (including the name of the issuing authority, the date of issue, and any identifying information). Permits should encompass collection and, where applicable, export.*
Specimen deposition	*Indicate where the specimens have been deposited to permit free access by other researchers.*
Dating methods	*If new dates are provided, describe how they were obtained (e.g. collection, storage, sample pretreatment and measurement), where they were obtained (i.e. lab name), the calibration program and the protocol for quality assurance OR state that no new dates are provided.*

☐ Tick this box to confirm that the raw and calibrated dates are available in the paper or in Supplementary Information.

Ethics oversight	*Identify the organization(s) that approved or provided guidance on the study protocol, OR state that no ethical approval or guidance was required and explain why not.*

Note that full information on the approval of the study protocol must also be provided in the manuscript.

Animals and other research organisms

Policy information about studies involving animals; ARRIVE guidelines recommended for reporting animal research, and Sex and Gender in Research

Laboratory animals	C57BL/6JGpt mice (Strain NO. N000013), CD45.1 congenic mice (C57BL/6JGpt-Ptprcem1Cin(p.K302E)/Gpt, Strain No. T054816), Rag1–/– mice (C57BL/6JGpt-Rag1em1Cd3259/Gpt, Strain NO. T004753) involved in this study were purchased from GemPharmatech (Nanjing, China). FOXP3YFP mice (B6.129(Cg)-Foxp3tm4(YFP/icre) Ayr/J, Strain #016959) were purchased from Jackson laboratory. All mice were bred and maintained under specific pathogen-free (SPF) conditions in the animal care facility at China Medical University. Sex- and age-matched mice were used. All mice were used at the age of 7–8 weeks. Littermate mice were selected if possible.
Wild animals	This study involved no wild animals.
Reporting on sex	Littermate mice were paired according to sex. Each pair contained male or female mice only.
Field-collected samples	This study involved no field-collected samples.
Ethics oversight	The experimental protocols were approved by the Institutional Animal Care and Use Committees (IACUC) of China Medical University and all animal experiments were performed in accordance with the National Institute of Health Guide for the Care and Use of Laboratory Animals (NO. CMU2022229).

Note that full information on the approval of the study protocol must also be provided in the manuscript.

Clinical data

Policy information about clinical studies

All manuscripts should comply with the ICMJE guidelines for publication of clinical research and a completed CONSORT checklist must be included with all submissions.

Clinical trial registration	*Provide the trial registration number from ClinicalTrials.gov or an equivalent agency.*
Study protocol	*Note where the full trial protocol can be accessed OR if not available, explain why.*

Data collection	*Describe the settings and locales of data collection, noting the time periods of recruitment and data collection.*
Outcomes	*Describe how you pre-defined primary and secondary outcome measures and how you assessed these measures.*

Dual use research of concern

Policy information about dual use research of concern

Hazards

Could the accidental, deliberate or reckless misuse of agents or technologies generated in the work, or the application of information presented in the manuscript, pose a threat to:

No	Yes	
☐	☐	Public health
☐	☐	National security
☐	☐	Crops and/or livestock
☐	☐	Ecosystems
☐	☐	Any other significant area

Experiments of concern

Does the work involve any of these experiments of concern:

No	Yes	
☐	☐	Demonstrate how to render a vaccine ineffective
☐	☐	Confer resistance to therapeutically useful antibiotics or antiviral agents
☐	☐	Enhance the virulence of a pathogen or render a nonpathogen virulent
☐	☐	Increase transmissibility of a pathogen
☐	☐	Alter the host range of a pathogen
☐	☐	Enable evasion of diagnostic/detection modalities
☐	☐	Enable the weaponization of a biological agent or toxin
☐	☐	Any other potentially harmful combination of experiments and agents

Plants

Seed stocks	*Report on the source of all seed stocks or other plant material used. If applicable, state the seed stock centre and catalogue number. If plant specimens were collected from the field, describe the collection location, date and sampling procedures.*
Novel plant genotypes	*Describe the methods by which all novel plant genotypes were produced. This includes those generated by transgenic approaches, gene editing, chemical/radiation-based mutagenesis and hybridization. For transgenic lines, describe the transformation method, the number of independent lines analyzed and the generation upon which experiments were performed. For gene-edited lines, describe the editor used, the endogenous sequence targeted for editing, the targeting guide RNA sequence (if applicable) and how the editor was applied.*
Authentication	*Describe any authentication procedures for each seed stock used or novel genotype generated. Describe any experiments used to assess the effect of a mutation and, where applicable, how potential secondary effects (e.g. second site T-DNA insertions, mosiacism, off-target gene editing) were examined.*

ChIP-seq

Data deposition

☐ Confirm that both raw and final processed data have been deposited in a public database such as GEO.

☐ Confirm that you have deposited or provided access to graph files (e.g. BED files) for the called peaks.

Data access links<br>*May remain private before publication.*	*For "Initial submission" or "Revised version" documents, provide reviewer access links. For your "Final submission" document, provide a link to the deposited data.*
Files in database submission	*Provide a list of all files available in the database submission.*
Genome browser session<br>(e.g. UCSC)	*Provide a link to an anonymized genome browser session for "Initial submission" and "Revised version" documents only, to enable peer review. Write "no longer applicable" for "Final submission" documents.*

Methodology

Replicates	*Describe the experimental replicates, specifying number, type and replicate agreement.*
Sequencing depth	*Describe the sequencing depth for each experiment, providing the total number of reads, uniquely mapped reads, length of reads and whether they were paired- or single-end.*
Antibodies	*Describe the antibodies used for the ChIP-seq experiments; as applicable, provide supplier name, catalog number, clone name, and lot number.*
Peak calling parameters	*Specify the command line program and parameters used for read mapping and peak calling, including the ChIP, control and index files used.*
Data quality	*Describe the methods used to ensure data quality in full detail, including how many peaks are at FDR 5% and above 5-fold enrichment.*
Software	*Describe the software used to collect and analyze the ChIP-seq data. For custom code that has been deposited into a community repository, provide accession details.*

Flow Cytometry

Plots

Confirm that:

☒ The axis labels state the marker and fluorochrome used (e.g. CD4-FITC).

☒ The axis scales are clearly visible. Include numbers along axes only for bottom left plot of group (a 'group' is an analysis of identical markers).

☒ All plots are contour plots with outliers or pseudocolor plots.

☒ A numerical value for number of cells or percentage (with statistics) is provided.

Methodology

Sample preparation	Single-cell suspension preparation: Isolation of lymphocytes from mouse lung and spleen was in accordance with the guidance of flow cytometry (FC). In short, the lung was minced and digested with a digestion solution containing type I collagenase (2 mg/mL), DNase I (100U/mL), and complete media (DMEM plus 4% BSA). The suspension was incubated on a rocker at 37°C. Collagen-digested lungs were dispersed. Red blood cells were lysed using ACK lysis buffer. Leukocytes were enriched from the cell digestion using Percoll gradients (80%/40%) (Cytiva). Spleen cells were prepared by pressing the tissues through 70 µm cell strainers using the end of a sterile plunger of a 5 mL syringe. Single-cell suspension was prepared for subsequent flow cytometry staining or cell sorting.<br>Flow cytometry analysis: FC analysis was performed according to the guidance. Live/Dead cell viability dye Aqua (Invitrogen) was used to exclude the influence of dead cells. Fluorescently labeled antibodies to cell surface antigens were applied and incubated at 4°C for 30 min. For the intracellular transcriptional factor staining, cells were first stained for cell-surface markers and fixed with 4% paraformaldehyde, permeabilized with the FOXP3 transcription factor staining buffer set (eBioscience), and then stained with indicated antibodies to transcriptional factors at 4°C overnight. For the cytokine staining, leukocyte stimulation cocktail containing PMA, ionomycin, brefeldin A, and monensin (Invitrogen) was used to stimulate cells for 4 h before staining. The antibodies detecting transcriptional factors or cytokines were incubated at 4°C overnight. FC analysis was performed on BD FACS Celesta (BD Bioscience). FACS data were analyzed with Flowjo 10.6 software. The gating strategy is shown in Figure S1a. All involved anti-mouse antibodies are listed in Antibody section.
Instrument	Flow Cytometry: BD FACS Celesta (BD Biosciences)<br>Cell Sorting: BD FACSAria II (BD Biosciences)
Software	Flowjo 10.6 (BD Biosciences)
Cell population abundance	Sorted cells were over 95% pure, as determined by FACS reanalysis.
Gating strategy	The gating strategy of FC analysis was provided in Fig S1a, while the gating strategy of FACS sorting was displayed in Fig 5h.

☒ Tick this box to confirm that a figure exemplifying the gating strategy is provided in the Supplementary Information.

Magnetic resonance imaging

Experimental design

Design type	*Indicate task or resting state; event-related or block design.*
Design specifications	*Specify the number of blocks, trials or experimental units per session and/or subject, and specify the length of each trial or block (if trials are blocked) and interval between trials.*

Behavioral performance measures	*State number and/or type of variables recorded (e.g. correct button press, response time) and what statistics were used to establish that the subjects were performing the task as expected (e.g. mean, range, and/or standard deviation across subjects).*

Acquisition

Imaging type(s)	*Specify: functional, structural, diffusion, perfusion.*
Field strength	*Specify in Tesla*
Sequence & imaging parameters	*Specify the pulse sequence type (gradient echo, spin echo, etc.), imaging type (EPI, spiral, etc.), field of view, matrix size, slice thickness, orientation and TE/TR/flip angle.*
Area of acquisition	*State whether a whole brain scan was used OR define the area of acquisition, describing how the region was determined.*

Diffusion MRI ☐ Used ☐ Not used

Preprocessing

Preprocessing software	*Provide detail on software version and revision number and on specific parameters (model/functions, brain extraction, segmentation, smoothing kernel size, etc.).*
Normalization	*If data were normalized/standardized, describe the approach(es): specify linear or non-linear and define image types used for transformation OR indicate that data were not normalized and explain rationale for lack of normalization.*
Normalization template	*Describe the template used for normalization/transformation, specifying subject space or group standardized space (e.g. original Talairach, MNI305, ICBM152) OR indicate that the data were not normalized.*
Noise and artifact removal	*Describe your procedure(s) for artifact and structured noise removal, specifying motion parameters, tissue signals and physiological signals (heart rate, respiration).*
Volume censoring	*Define your software and/or method and criteria for volume censoring, and state the extent of such censoring.*

Statistical modeling & inference

Model type and settings	*Specify type (mass univariate, multivariate, RSA, predictive, etc.) and describe essential details of the model at the first and second levels (e.g. fixed, random or mixed effects; drift or auto-correlation).*
Effect(s) tested	*Define precise effect in terms of the task or stimulus conditions instead of psychological concepts and indicate whether ANOVA or factorial designs were used.*

Specify type of analysis: ☐ Whole brain ☐ ROI-based ☐ Both

Statistic type for inference<br>(See Eklund et al. 2016)	*Specify voxel-wise or cluster-wise and report all relevant parameters for cluster-wise methods.*
Correction	*Describe the type of correction and how it is obtained for multiple comparisons (e.g. FWE, FDR, permutation or Monte Carlo).*

Models & analysis

n/a	Involved in the study
☐	☐ Functional and/or effective connectivity
☐	☐ Graph analysis
☐	☐ Multivariate modeling or predictive analysis

Functional and/or effective connectivity	*Report the measures of dependence used and the model details (e.g. Pearson correlation, partial correlation, mutual information).*
Graph analysis	*Report the dependent variable and connectivity measure, specifying weighted graph or binarized graph, subject- or group-level, and the global and/or node summaries used (e.g. clustering coefficient, efficiency, etc.).*
Multivariate modeling and predictive analysis	*Specify independent variables, features extraction and dimension reduction, model, training and evaluation metrics.*
